# Tea leaf-derived exosome-like nanotherapeutics retard breast tumor growth by pro-apoptosis and microbiota modulation

**DOI:** 10.1186/s12951-022-01755-5

**Published:** 2023-01-04

**Authors:** Qiubing Chen, Menghang Zu, Hanlin Gong, Ya Ma, Jianfeng Sun, Susan Ran, Xiaoxiao Shi, Jinming Zhang, Bo Xiao

**Affiliations:** 1 State Key Laboratory of Silkworm Genome Biology, College of Sericulture, Textile and Biomass Sciences, Southwest University, Beibei, Chongqing, 400715 China; 2 Chongqing Key Laboratory of Soft-Matter Material Chemistry and Function Manufacturing, School of Materials and Energy, Southwest University, Beibei, Chongqing, 400715 China; 3 Department of Integrated Traditional Chinese and Western Medicine, West China Hospital, Sichuan University, Chengdu, 610041 Sichuan China; 4 Nuffield Department of Orthopedics, Rheumatology and Musculoskeletal Sciences, Botnar Research Centre, University of Oxford, Headington, OX3 7LD Oxford UK; 5 Loomis Chaffee School, Windsor, CT 06095 USA; 6 State Key Laboratory of Southwestern Chinese Medicine Resources, Pharmacy School, Chengdu University of Traditional Chinese Medicine, Chengdu, 611137 Sichuan China

**Keywords:** Apoptosis, Breast cancer, Intestinal microbiota rebalance, Natural nanomedicine, Reactive oxygen species

## Abstract

While several artificial nanodrugs have been approved for clinical treatment of breast tumor, their long-term applications are restricted by unsatisfactory therapeutic outcomes, side reactions and high costs. Conversely, edible plant-derived natural nanotherapeutics (NTs) are source-widespread and cost-effective, which have been shown remarkably effective in disease treatment. Herein, we extracted and purified exosome-like NTs from tea leaves (TLNTs), which had an average diameter of 166.9 nm and a negative-charged surface of − 28.8 mV. These TLNTs contained an adequate slew of functional components such as lipids, proteins and pharmacologically active molecules. In vitro studies indicated that TLNTs were effectively internalized by breast tumor cells (4T1 cells) and caused a 2.5-fold increase in the amount of intracellular reactive oxygen species (ROS) after incubation for 8 h. The high levels of ROS triggered mitochondrial damages and arrested cell cycles, resulting in the apoptosis of tumor cells. The mouse experiments revealed that TLNTs achieved good therapeutic effects against breast tumors regardless of intravenous injection and oral administration through direct pro-apoptosis and microbiota modulation. Strikingly, the intravenous injection of TLNTs, not oral administration, yielded obvious hepatorenal toxicity and immune activation. These findings collectively demonstrate that TLNTs can be developed as a promising oral therapeutic platform for the treatment of breast cancer.

**Graphic Abstract:**

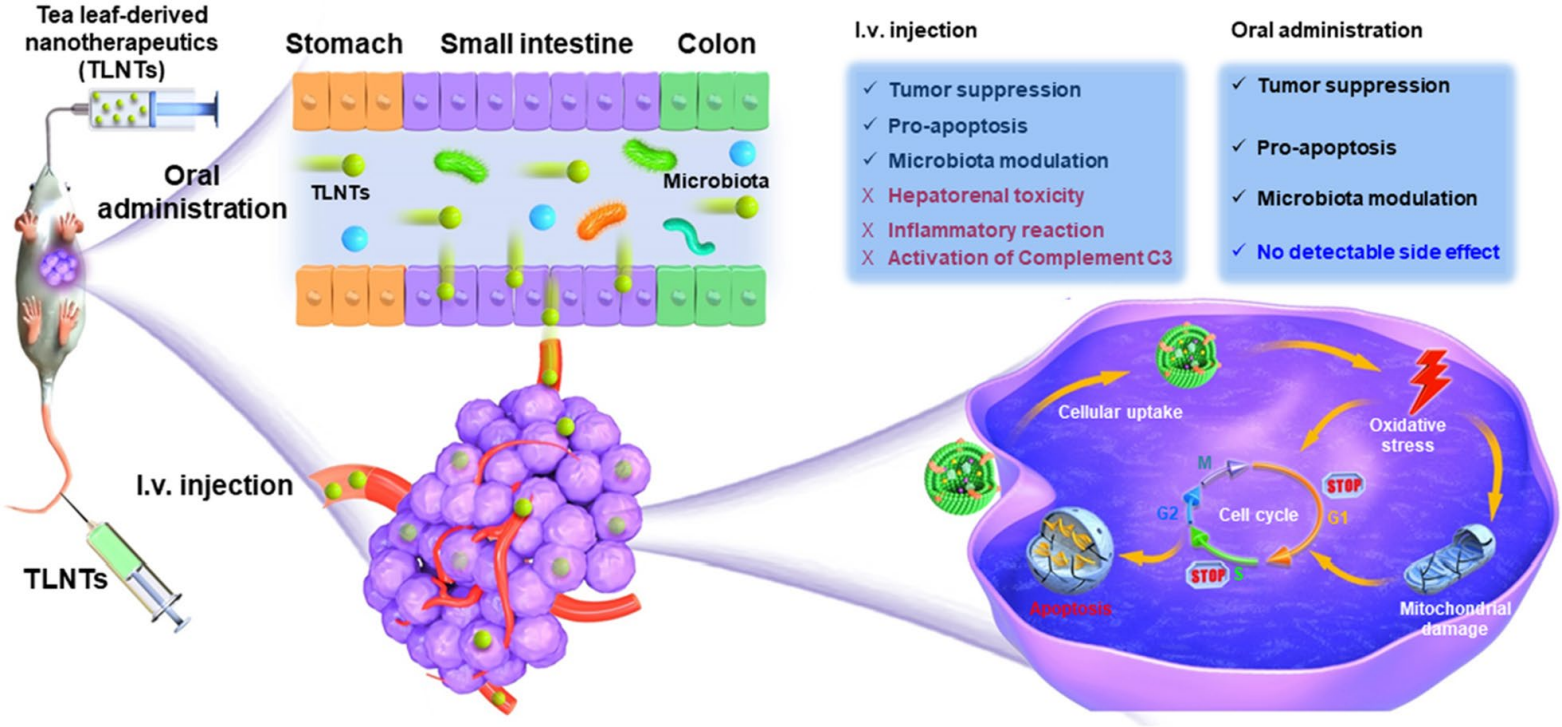

**Supplementary Information:**

The online version contains supplementary material available at 10.1186/s12951-022-01755-5.

## Introduction

Breast cancer is the most common carcinoma in female, which is one of the principal causes of cancer-related mortality worldwide [1]. Currently, surgery, chemotherapy and radiotherapy lie in the central tenet of treatment strategies for breast cancer, which have been shown effective in eliminating or retarding the growth of breast tumors [2]. A number of inherent obstacles in limiting their biomedical applications cannot be neglected. These include (1) poor compliance in clinic as well as re-growth and metastasis of residual tumor cells after surgery [3], (2) serious systemic adverse effects (*e.g.*, nausea, vomiting and immunosuppression) after radiotherapy [4], and (3) undesirable therapeutic outcomes and unwanted side effects after chemotherapy [5]. Therefore, there is a dire need for exploring innovative therapeutic strategies to obtain satisfactory anti-breast cancer effects.

Nanomedicines have attracted a wealth of interest across clinical scientific communities by demonstrating promising results in the treatment of various diseases, including cancers, alcoholic livers, inflammatory disorders and COVID-19 [6–8]. Unfortunately, very few nanotherapeutics (NTs) have been approved for applications in clinical practice, likely due to their low long-term treatment efficacies, potential adverse toxicity to the healthy tissues, arduous mass-production and residuals of organic solvents and toxic catalysts [9, 10]. Recently, scientists have extensively appealed for the adoption of “industry-style frameworks” with a particular focus on patients and their diseases from the beginning (“disease first”) instead of chemistry and material science (“formulation first”) for the development of nanomedicines [11]. To facilitate the development of nanomedicines for clinical translation, we chose to test natural NTs that could be massively obtained from edible plants (*e.g.*, blueberry, ginger and tea flower) via green extraction methods [9, 12, 13]. These natural NTs contained abundant bio-functional molecules (*e.g.*, glycolipids, polysaccharides and proteins) to exert powerful anti-inflammatory, anti-oxidant and anti-tumor activities. For instance, Yang et al*.* isolated NTs from lemon juice using the electrophoretic technique. They found that these NTs could increase the concentrations of intracellular reactive oxygen species (ROS) and induce S-phase cell cycle arrest, leading to the apoptosis of tumor cells both in vitro and in vivo [14]. Later, Cao and colleagues extracted NTs from ginseng based on differential centrifugation accompanied by density gradient centrifugation. The obtained NTs had the capacity to induce M1 polarization of macrophages through Toll-like receptor-4 and myeloid differentiation antigen 88-mediated signaling, produce large amounts of ROS in the cytoplasm and retard tumor growth in mice bearing subcutaneous melanoma tumors [15]. Tea ranks as the second most abundant drink due to its flavors and healthy ingredients. For instant, epigallocatechin gallate (EGCG) is the main polyphenol in tea leaves, which has numerous health beneficial effects, including anticarcinogenesis, antisepsis and anti-inflammation [16–19]. Very recently, our group made the first attempt to extract and purify NTs from tea leaves, and we further demonstrated that these natural NTs could prevent colitis-associated orthotopic colon cancer by reducing pro-inflammatory cytokine levels, restoring disrupted colonic barriers and improving gut microbiota diversity [10]. However, it is still unclear as to whether these NTs are able to inhibit the parenteral tumors via the intravenous (i.v.) injection or oral administration.

**Scheme 1.**
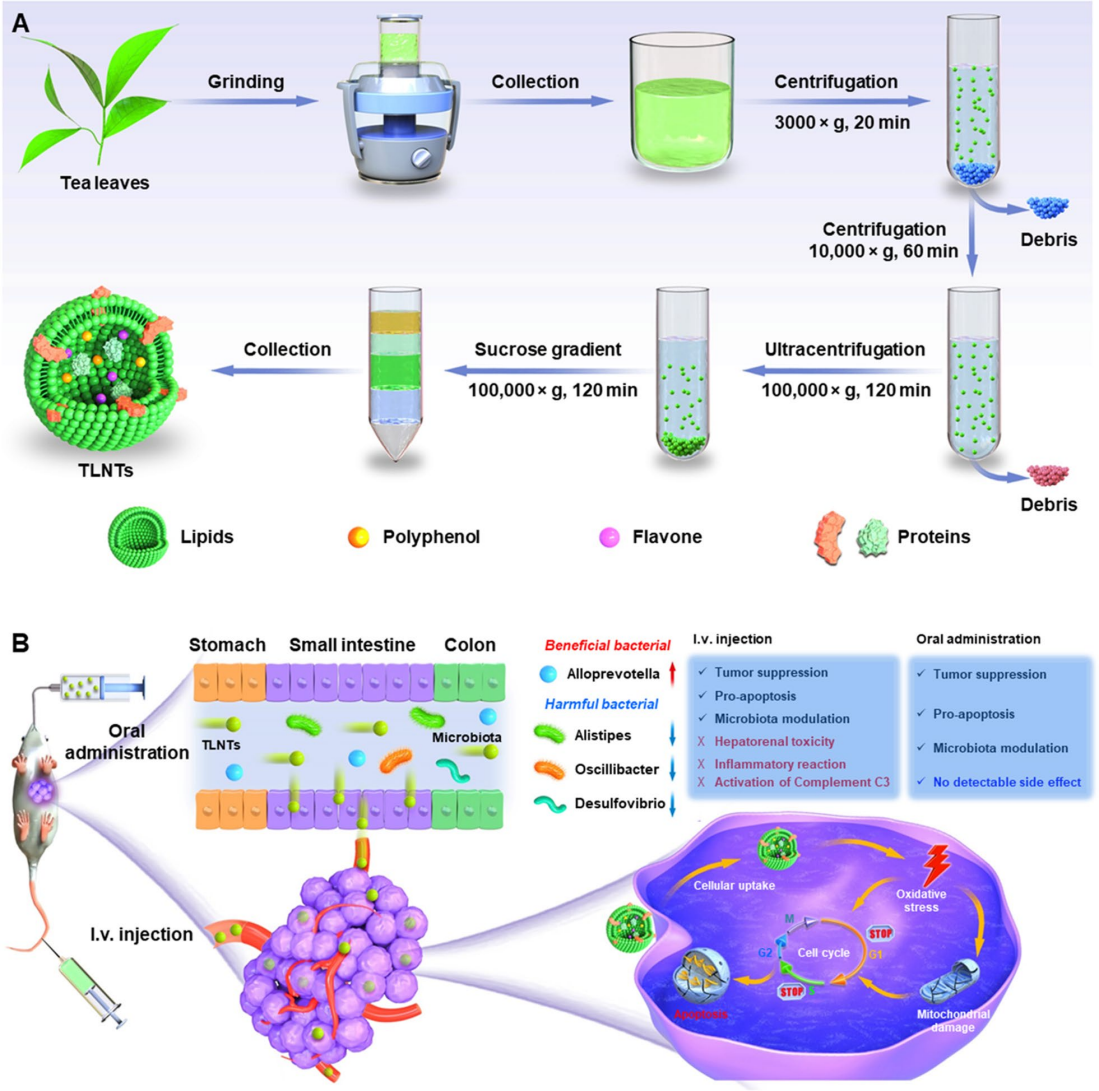
Schematic diagram of the extraction and purification processes of TLNTs and their application in the treatment of breast cancer via i.v. injection and oral administration. **A** The production processes of TLNTs based on differential centrifugation accompanied by density gradient centrifugation. **B** The in vivo anti-tumor mechanism and comparative therapeutic outcomes of TLNTs against breast cancer via i.v. injection and oral administration

In the current study, we isolated natural NTs from fresh tea leaves, characterized their stabilities in the gastrointestinal tract (GIT) and determined their contents such as lipids, proteins, polyphenols and flavonoids (Scheme 1A). Subsequently, we evaluated their in vitro anti-tumor effects against various tumor cell lines and uncovered the underlying anti-tumor mechanism in the light of the intracellular ROS generation, damages to mitochondria and cell cycle arrest. Finally, we comparatively assessed in vivo bio-distributions, therapeutic outcomes against breast cancer, impacts on gene expression profiles and intestinal microbiota and in vivo biosafety of the tea leaf-derived NTs (TLNTs) after i.v. injection and oral administration (Scheme 1B).

## Materials and methods

### Materials

Fresh tea leaves (Yongchuan Xiuya) were purchased from Chongqing Ersheng Tea Co., Ltd. (Chongqing, China). Sucrose, dimethyl sulfoxide (DMSO) and Triton X-100 were obtained from Aladdin (Shanghai, China). Antibiotics (ATBs), 1,1ʹ-dioctadecyl-3,3,3ʹ,3ʹ-tetramethylindotricarbocyanine iodide (DiR), 3,3ʹ-dioctadecyloxacarbocyanine perchlorate (DiO) and rhodamine B-labeled phalloidin were supplied by Promokine (Heidelberg, Germany). 2-(4-Amidinophenyl)-6-indolecarbamidine dihydrochloride (DAPI), methylthiazolyldiphenyl-tetrazolium bromide (MTT), ROS assay kits, cell cycle analysis kits, enhanced mitochondrial membrane potential assay kits with JC-1 assay kits, BCA protein assay kits, Hoechst 33342, Annexin V-FITC apoptosis detection kits and terminal deoxynucleotidyl transferase-mediated dUTP-biotin nick end labeling (TUNEL) apoptosis assay kits were purchased from Beyotime Institute of Biotechnology (Shanghai, China). Anti-GAPDH, anti-Cyclin A, anti-Cyclin B, and anti-Cyclin D antibodies were supplied by Novus Biologicals (Littleton, CO, USA). Enzyme-linked immunosorbent assay (ELISA) kits (TNF-α, IL-6 and IL-12) were purchased from Beijing solarbio science & technology Co., ltd (Beijing, China). Complement 3 assay kits, alanine aminotransferase assay kits (ALT), aspartate aminotransferase assay kits (AST), urea nitrogen activity assay kits (BUN) and creatinine assay kits (CRE) were from Nanjing Jiancheng Bioengineering Institute (Jiangsu, China).

### Isolation and purification of TLNTs

Tea leaves were initially washed with water, deposited in cold phosphate buffered saline (PBS, pH 7.4) and homogenized at a high speed for 10 min in a blender (Midea, China). The resulted juice was centrifuged at 1000 × *g* for 20 min and 5000 × *g* for 40 min to eliminate the large plant debris. Thereafter, TLNTs were purified by sucrose density gradient ultra-centrifugation (8, 30, 45 and 60% sucrose in 20 mM Tris-Cl, pH 7.2) at 120,000 × *g* for 2 h. Eventually, we collected TLNTs from the 30/45% sucrose interface. Their protein concentrations were quantified using a BCA assay kit, and the acquired TLNTs were deposited at − 80 °C for further applications.

### Cell culture

The mouse colon carcinoma cell line (CT-26), human breast cancer cell line (MCF-7 cell) and mouse breast cancer cell line (4T1) were supplied by the Cell Bank of the Chinese Academy of Sciences (Shanghai, China). These cells were cultured in Dulbecco’s modified eagle medium (DMEM) containing fetal bovine serum (10%, v/v) and penicillin/streptomycin (1%, w/v) in a CO_2_ incubator at 37 °C.

### In vitro anti-tumor activity of TLNTs

In vitro anti-tumor activities of TLNTs were determined by the MTT assay. CT-26 cells, MCF-7 cells and 4T1 cells were cultured in 96-well plates at a density of 1 × 10^4^ cells/well and incubated overnight. They were incubated with different amounts of TLNTs (protein concentrations: 0.5, 1, 2, 4, 8, 16, 32 and 64 μg/mL) in a serum-free medium for 24 and 48 h, respectively. The TLNT-contained medium was removed afterwards. Cells were washed 3 times with PBS. Cells were incubated with 100 μL of MTT (0.5 mg/mL) at 37 °C for 4 h until a purple precipitate was visible. Thereafter, the media were discarded, and DMSO (100 μL) was added to each well prior to spectrophotometric measurements at 570 nm.

### In vitro pro-apoptosis property of TLNTs

The annexin V-FITC/propidium iodide (PI) apoptosis assay was carried out to evaluate the pro-apoptosis properties of TLNTs. 4T1 cells were incubated in 6-well culture plates at a density of 1 × 10^5^ per well and incubated overnight. Thereafter, cells were co-cultured with 16 μg/mL TLNTs for 4 and 8 h, respectively. At the end of the co-incubation, cells were washed 3 times with cold PBS, digested by trypsin and collected by centrifugation at 1000 × *g* for 3 min. Cells were suspended in annexin V binding buffer, and the obtained cell suspension (100 μL) was transferred to a 2 mL culture tube containing annexin V-FITC and PI. After co-incubation at 37 °C for 20 min in the dark, the annexin V binding buffer (400 μL) was added to each tube. Finally, cells were analyzed by flow cytometry (FCM, Beckman Coulter Inc, USA).

### Impacts of TLNTs on mitochondrial membrane potential

A fluorescent probe (JC-1) was utilized to estimate the variations of the mitochondrial membrane potential after the treatment of TLNTs. Briefly, 4T1 cells were seeded in 24-well plates at a density of 1 × 10^5^ cells/well and incubated overnight. Thereafter, cells were co-cultured with TLNTs (16 μg/mL) for 4 h and further co-incubated in JC-1 solution for 30 min at 37 °C. Finally, cells were incubated with Hoechst 33,342 for 5 min and image-processed using a high-resolution laser confocal microscope (CLSM, Olympus, FV-3000, Japan).

### Impacts of TLNTs on intracellular ROS generation

Intracellular ROS was detected by using an oxidation-sensitive fluorescent probe (DCFH-DA). Briefly, 4T1 cells were seeded in 24-well plates at a density of 1 × 10^5^ cells/well and incubated overnight. After co-incubation with TLNTs (16 µg/mL) for 4 h, cells were washed 3 times in cold PBS. Thereafter, cells were incubated with DCFH-DA (10 µM) at 37 °C for 20 min, washed 3 times with PBS and incubated in Hoechst 33342 solution for 5 min. Finally, cells were incubated with Hoechst 33342 for 5 min and image-processed using a CLSM (Olympus, FV-3000, Japan).

### Impacts of TLNTs on cell cycle

FCM was used to assess the cell cycle phases of 4T1 cells receiving the treatment of TLNTs for 12 and 24 h, respectively. In brief, 4T1 cells were cultured in 6-well plates (5 × 10^5^ cell/well). After overnight incubation, cells were co-cultured with TLNTs (protein concentration: 16 μg/mL) for 12 and 24 h, respectively. Subsequently, cells were digested by trypsin, collected by centrifugation, washed 3 times with cold PBS, fixed in 70% ethanol (4 °C) and treated with PI and RNase A for 30 min. Finally, cells were analyzed by FCM (Beckman Coulter Inc, USA). Additionally, to evaluate the cell cycle inhibition mechanism, western blotting was used to determine the relative expression levels of Cyclin A, Cyclin B and Cyclin D.

### In vivo bio-distribution of TLNT*s*

6-Week-old female BALB/c nude mice were obtained from Chongqing Leibitt Biotechnology Co. Ltd. (Chongqing, China). Mice investigations were approved by the Institutional Animal Care and Use Committee of Southwest University. To track in vivo bio-distribution of TLNTs, a near-infrared fluorescence probe (DiR) was used to label these TLNTs. 4T1 cells (5 × 10^6^) were injected in the mammary glands. When the tumor volume reached 100 mm^3^, mice were administered with DiR-TLNTs at an equivalent DiR concentration (3 mg protein/kg per mouse) via i.v. injection or oral route. At predetermined time points (6, 12, 24 and 48 h), mice were euthanized. The major organs, tumors and GITs were isolated, and their fluorescence images were made using an IVIS spectrum imaging system (PerkinElmer/Caliper LifeSciences, Hopkinton, MA, USA).

### In vivo anti-tumor effects of TLNTs

The subcutaneous breast tumor mouse model was established according to the protocol mentioned above. When tumor volume reached 100 mm^3^, mice were divided into 7 groups: the control group, the TLNT (i.v., low)-treated group, the TLNT (i.v., high)-treated group, the TLNT (i.v., high, ATB)-treated group, the TLNT (oral, low)-treated group, the TLNT (oral, high)-treated group and the TLNT (oral, high, ATB)-treated group, respectively. Mice were administrated with 1.5 (low) or 3 (high) mg protein/kg mice of TLNTs (100 μL) in the presence or absence of ATBs in the drinking water. Mice were administrated with TLNTs every other day for a total of 4 doses. Mice body weights and tumor volumes were recorded. At the end of the experiment, mice were sacrificed, and tumors were collected, fixed in paraformaldehyde solution (4%, v/v) and embedded in paraffin. The tissues were sectioned (5 µm) and stained with hematoxylin and eosin (H&E), Ki67 and TUNEL kits, respectively. To investigate the anti-tumor mechanism of TLNTs, the transcriptome of tumor tissue was performed. The major operation during statistical analysis is that raw data were stringently filtered to ensure the quality, and the up-regulated and down-regulated genes were identified and mapped with a fold change ≥ 2 and *p*-value < 0.05.

### Statistical analysis

Data were presented as means ± standard error of the mean (S.E.M.). Statistical analysis was carried out using Student’s *t*-test or one-way ANOVA. Statistical significance was represented by *p < 0.05, **p < 0.01, and ***p < 0.001.

## Results and discussion

### Physicochemical characterization of TLNTs

**Fig. 1:**
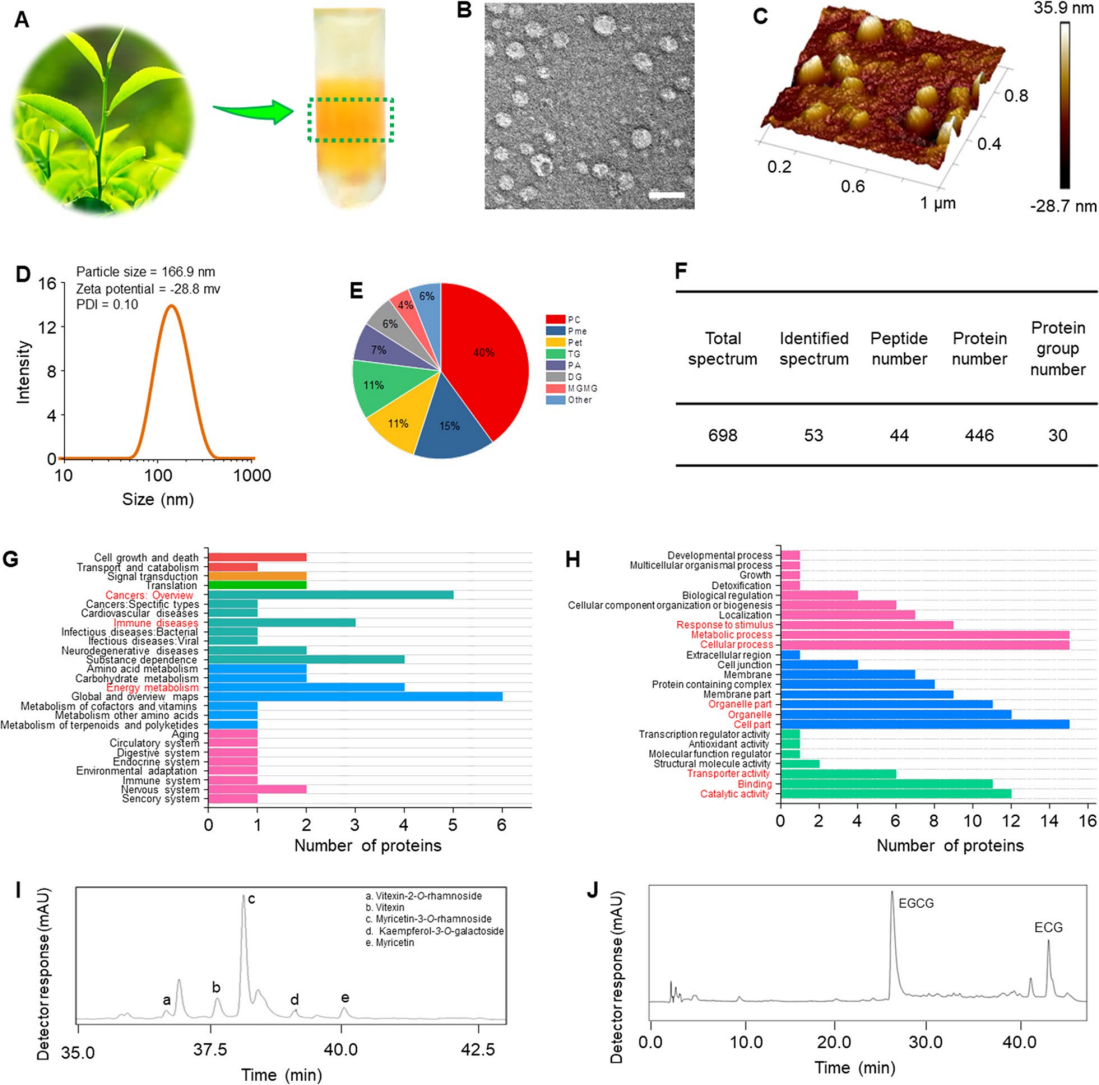
Physicochemical and functional characterizations of TLNTs. **A** TLNTs in the sucrose gradients after ultracentrifugation. **B** TEM imaging (scale bar: 100 nm), **C** AFM imaging, **D** hydrodynamic particle size distribution, **E** lipid compositions, **F** protein summary, **G** KEGG annotated statistical charts and **H** Go secondary classification statistical charts of TLNTs. **I** Flavonoids and **J** polyphenols in TLNTs. *EGCG* epigallocatechin gallate, *ECG* epicatechin gallate

TLNTs were extracted from the juice of fresh tea leaves by differential centrifugation and further purified by sucrose density gradient ultracentrifugation. Driven by the sucrose gradient, TLNTs were mostly present at the interface of 30/45% (Fig. 1A). The transmission electron microscopy (TEM) and the atomic force microscopy (AFM) were employed to detect the morphology of the purified TLNTs. As shown in Fig. 1B, C, these NTs presented exosome-like spherical particles with an average particle size of 70 nm. The further dynamic light scattering (DLS) results revealed that TLNTs had a hydrodynamic particle size of 166.9 nm, a uniform size distribution (PDI = 0.100) and a negatively charged surface (− 28.8 mV), as shown in Fig. 1D. The difference among particle sizes determined by the TEM, AFM and DLS might be ascribed to the phenomenon that TLNTs are fully dehydrated prior to applying them for imaging, while they stay in highly swollen and wet states during the DLS test.

The lipidomic analysis implied that TLNTs were primarily composed of phosphatidylcholine (PC, 40%), phosphatidylmethanol (Pme, 15%), phosphatidylethanol (Pet, 11%), triglyceride (TG, 11%), phosphatidic acid (PA, 7%), diacylglycerol (DG, 6%) and monogalactosyldiacyglycerol (MGMG, 4%) (Fig. 1E). All these molecules were amphiphilic, and they established the structural foundation for stability of TLNTs. Furthermore, protein compositions in TLNTs were investigated by using liquid chromatography coupled with tandem mass spectrometry assays. It was found that 446 kinds of proteins were present in TLNTs (Fig. 1F). The biological functions of these proteins were analyzed by using the Gene Ontology (GO) database. We found that over 40 kinds of proteins related to cancers, metabolic processes, immune diseases and cell components (Fig. 1G). According to the GO database and Genes and Genomes (KEGG) Annotation Path Analysis, TLNTs contained 11 kinds of proteins associated with cellular processes, metabolic processes, cell parts and catalytic activity (Fig. 1H).

Tea leaves are enriched with active small molecular constituents, such as polyphenols and flavonoids, which possess the capacity to impact on cell apoptosis, cell migration and immune responses [20–22]. Therefore, we quantified the contents of the active small molecules in TLNTs by high performance liquid chromatography-tandem mass spectrometry (HPLC-MS/MS). As presented in Fig. 1I, J, TLNTs contained large amounts of the well-documented anti-tumor polyphenols and flavonoids, including EGCG [23], vitexin-2-*O*-rhamnoside [24], vitexin [25], myricetin-3-*O*-rhamnoside [26], kaempferol-3-*O*-galactoside [27] and myricetin [28]. The results relevant to the TLNT compositions collectively provide a primary foundation for their application in cancer treatment.

### In vitro cellular uptake and anti-tumor activities of TLNTs

Efficient cell internalization of NTs is a prerequisite for exerting anti-tumor effects. The CLSM images revealed that the control cells (without NT treatment) exhibited no green fluorescence signals. On the contrary, after co-incubation for 5 h, almost all the cells showed obvious green signals (TLNTs), which were predominantly distributed in the cytoplasm (Additional file 1: Fig. S1). Furthermore, the cellular uptake efficiencies were quantified by FCM. It was observed that both cellular uptake percentages and mean fluorescence intensities (MFIs) of DiO-labeled TLNTs increased over time (Additional file 1: Fig. S2), and more than 80% of cells internalized TLNTs after 5-h co-incubation. These observations demonstrate that TLNTs are preferentially internalized by 4T1 cells and mainly present in the cytoplasm.

**Fig. 2:**
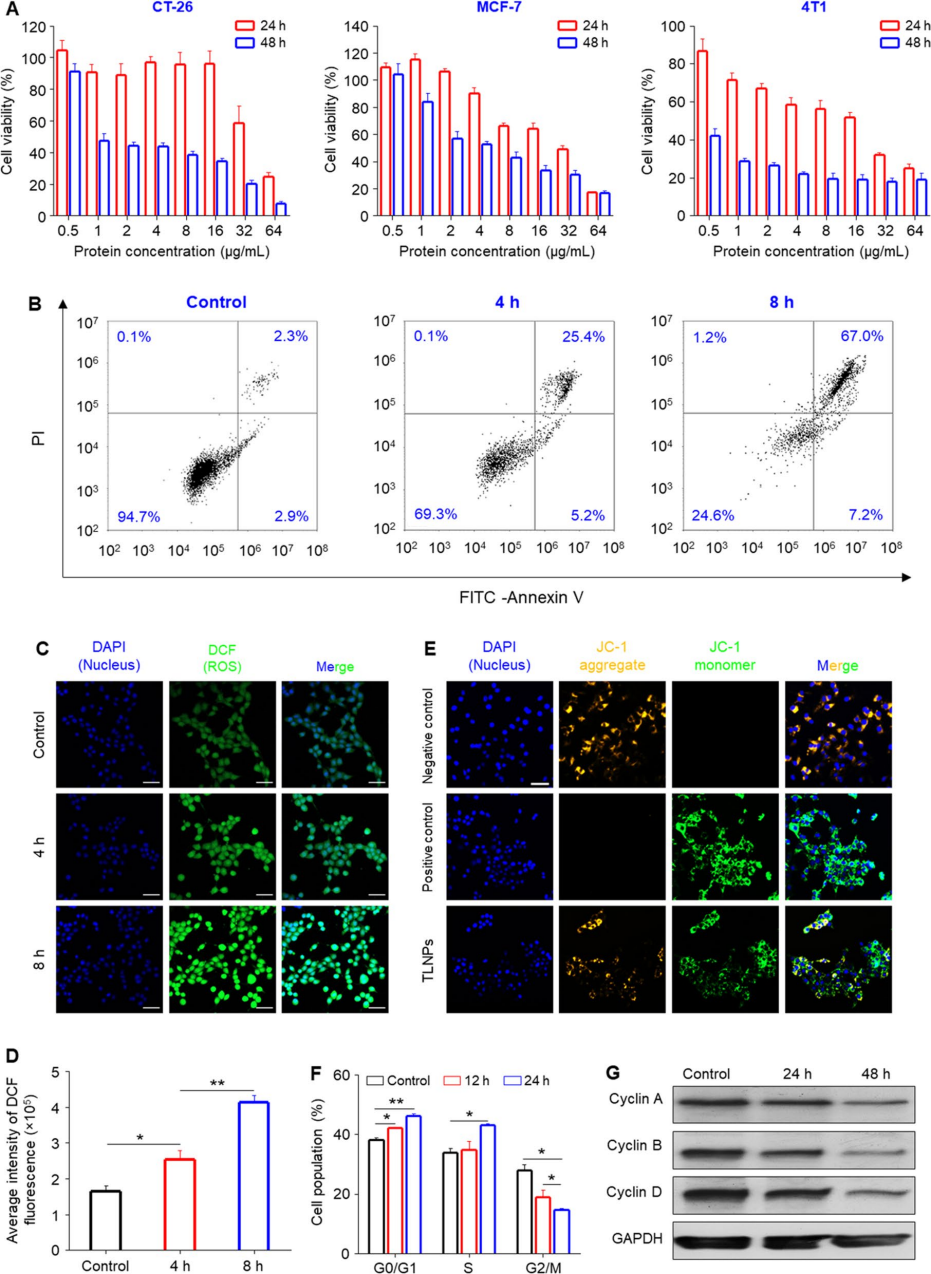
In vitro anti-tumor effects of TLNTs. **A** Cytotoxicity of TLNTs against various tumor cell lines after co-incubation with TLNTs at protein concentrations from 0.5 to 64 µg/mL for 24 and 48 h, respectively. Each point represents the mean ± S.E.M. (n = 5). **B** Pro-apoptotic properties of TLNTs after co-incubation with TLNTs for 4 and 8 h, respectively. **C** CLSM images of 4T1 cells stained with DCFH-DA after co-incubation with TLNTs for 4 and 8 h, respectively (scale bar: 50 μm). **D** ROS fluorescence intensity of 4T1 cells after co-incubation with TLNTs for 4 and 8 h, respectively. **E** Mitochondrial membrane potential changes in 4T1 cells (scale bar: 50 μm). **F** TLNTs restrained cell cycle progression in 4T1 cells after co-incubation with TLNTs for 12 and 24 h, respectively. Populations of 4T1 cells in various cell cycle phases were determined by FCM. Each point represents the mean ± S.E.M. (n = 3; *p < 0.05 and **p < 0.01). **G** Western blot analysis of 4T1 cells receiving the treatment of TLNTs for 48 h. Cyclin A, cyclin B and cyclin D proteins were probed. GAPDH was probed to ensure the equal loading of total proteins in each lane

Subsequently, the in vitro inhibitory effects of TLNTs on the proliferations of various tumor cell lines were investigated by the MTT assay. After co-incubation with TLNTs, the viabilities of CT-26 cells, MCF-7 cells and 4T1 cells gradually decreased with the increased TLNT concentrations and prolonged incubation (Fig. 2A). Meanwhile, we calculated the half maximal inhibitory concentrations (IC_50_) of TLNTs to compare the anti-proliferatory capacities of TLNTs (Additional file 1: Table S1). It was detected that after co-incubation for 24 h, the IC_50_ value of TLNTs against CT-26 cells was 1.2 and 1.8-fold higher than those against MCF-7 cells and 4T1 cells, respectively, and strikingly, became 2.5- and 220.8-fold higher, just forty-eight hours after incubation. These findings clearly demonstrate that TLNTs have a stronger capacity to suppress the proliferation of breast tumors (especially for 4T1 cells) than that of colon tumors. Therefore, TLNTs were used to inhibit the growth of breast tumor cells (4T1 cells). Subsequently, the pro-apoptotic properties of TLNTs against 4T1 cells were assessed by FCM. As shown in Fig. 2B, the apoptotic percentage of the control cells (without TLNT treatment) was 2.3%, whereas the apoptotic percentages of cells receiving the treatment of TLNTs for 4 and 8 h increased to 25.4% and 67.0%, respectively. It is widely believed that tumor metastasis is the main cause of death, and its fundamental step is tumor cell migration [29]. As a cheap and highly reproducible method, the cell scratch assay was performed to determine the suppressive effect of TLNTs on tumor cell migration. As seen in additional file 1: Fig. S3, the treatment of TLNTs significantly inhibited the migration of 4T1 cells, in comparison with the control cells.

### In vitro anti-tumor mechanism of TLNTs

Previous studies indicated that polyphenols and flavonoids could increase oxidative stress in tumor cells [30, 31]. Accordingly, we determined the produced amounts of intracellular ROS in the TLNT-treated cells. It was observed that after the TLNT treatment, green fluorescence signals gradually increased, and they were mainly present in the interior of the entire cells, including nucleus (Fig. 2C, D). These observations imply that large amounts of intracellular ROS are produced in the TLNT-treated cells, which might damage the fundamental substances involved in life activities of tumor cells [32, 33]. Mitochondria play important roles in various cell activities such as energy supply, metabolism and apoptosis [34]. To determine the damage effect of TLNTs on mitochondria, the mitochondrial membrane potential of the TLNT-treated 4T1 cells was evaluated using a mitochondrial probe (JC-1). Figure 2E showed that the negative control cells exhibited dark yellow fluorescence signals, indicating that these cells had largely intact mitochondrial membranes, while the positive control cells and the TLNT-treated cells appeared green, which suggested their damaged mitochondrial membranes, resulting in the decreased mitochondrial membrane potential. In all, these results demonstrate that TLNTs can efficiently induce mitochondrial damages.

Furthermore, we performed PI staining to investigate whether TLNTs could induce cell cycle arrest. It was determined in Fig. 2F that the percentages of the TLNT-treated 4T1 cells in G0/G1 phase and S phase remarkably increased, and those in G2/M phase greatly decreased. These results indicated that the TLNT treatment could block DNA replication in tumor cells and eventually cause cell death. The progression of cell cycle is regulated by various cycle proteins, including Cyclin A, Cyclin B and Cyclin D. These three kinds of proteins are involved in S/G2, G2/M and G1/S transitions of cell cycles, respectively. It was discovered that the intracellular amounts of Cyclin A, Cyclin B and Cyclin D were greatly decreased with the treatment of TLNTs, and the decreased trend was positively correlated with the treatment time (Fig. 2G), suggesting that TLNTs could inhibit cell cycle progression through down-regulation of the critical cycle proteins.

### In vivo bio-distribution of TLNTs

**Fig. 3:**
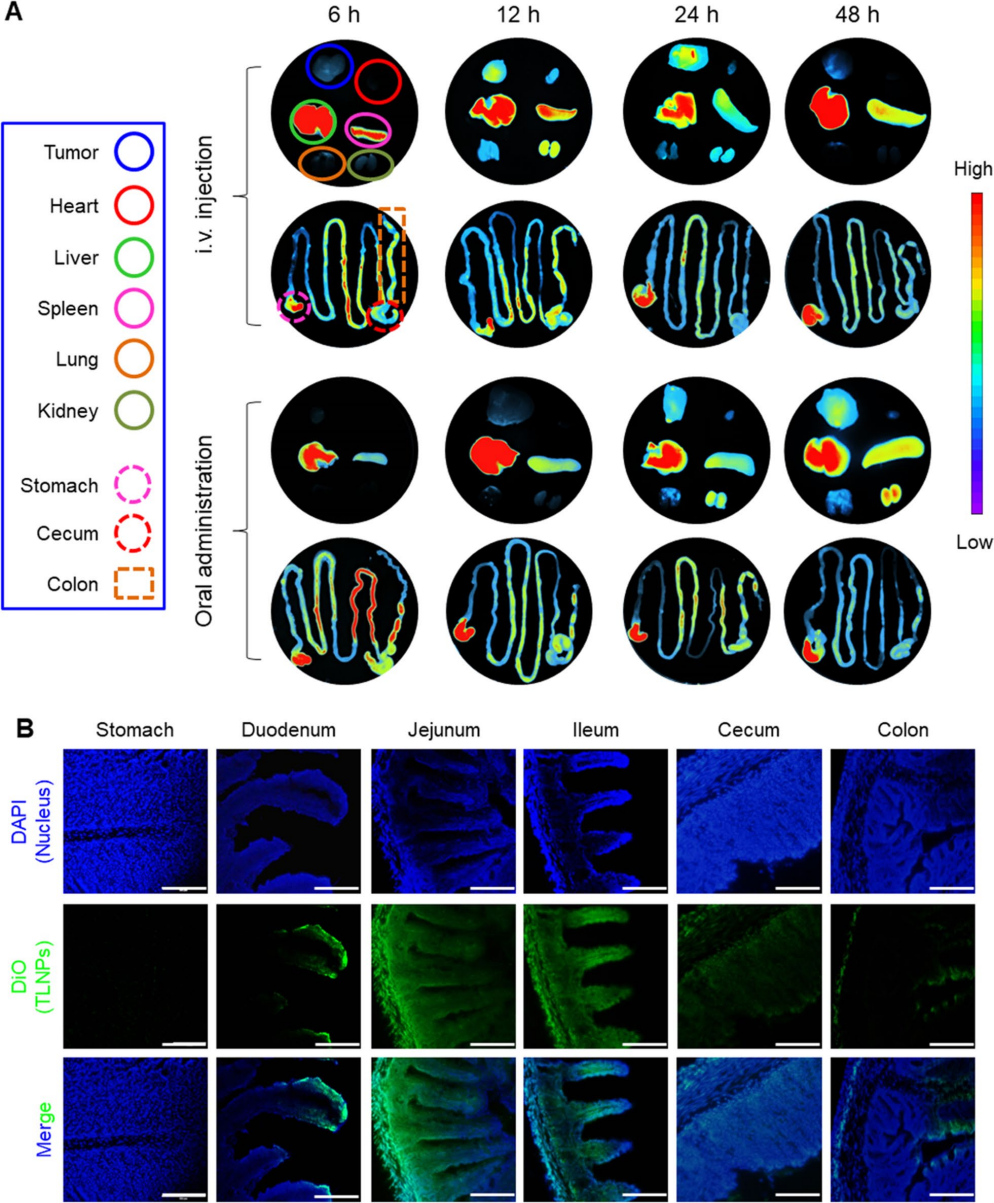
In vivo bio-distribution profiles of TLNTs. **A** Fluorescence images of tumors, five major organs (heart, liver, spleen, lung and kidney) and the GIT from breast tumor-bearing mice receiving the treatment of DiR-TLNTs via i.v. injection and oral administration at different time points (6, 12, 24 and 48 h). **B** Distribution profiles of TLNTs in different sections of the GIT following oral administration of DiO-labeled TLNTs for 6 h (scale bar: 100 µm)

I.v. injection is a widely used drug administration route in the preclinical treatment of various cancers, while alternatively, oral administration is the most preferable approach for patients in terms of safety, noninvasiveness, satisfactory compliance and cost-effectiveness [35]. As these two approaches possessed their own merits, we comparatively investigated the in vivo bio-distribution profiles of TLNTs via i.v. injection and oral administration. Initially, TLNTs were labeled with a DiR, administrated to mice bearing 4T1 tumors and processed by the IVIS spectrum imaging system. To determine whether fluorescence dye could remain in the TLNTs during the passage through the acidic stomach, we investigated its release profile from TLNTs. It was found that less than 20% of fluorescence dye was released from TLNTs after 6-h incubation in the stomach stimulating solution (pH 2.5) (Additional file 1: Fig. S4). As shown in Fig. 3A, TLNTs gradually accumulated in the tumors irrespective of drug administration approaches. We further found that the strongest fluorescence intensity of tumors after i.v. injection and oral route was readily found at the time point of 24 and 48 h, respectively (Additional file 1: Fig. S5). Nevertheless, the maximal fluorescence signals were present in the small intestine at the time point of 6 h for both administration routes, and then these signals tended to fade away.

To clarify the adsorption sites of TLNTs in the GIT after oral administration, mice were gavaged with DiO-labeled TLNTs (3 mg protein/kg mice), and their GITs were excised, sectioned and stained with DAPI. As visualized in Fig. 3B, there were a few green fluorescence signals in the mucosa of stomachs, ceca and colons, which appeared in the surface layer of the duodenum mucosa. It was worth noting that strong green signals suffused the mucosa of the jejunum and ileum, demonstrating that TLNTs might be adsorbed into the circulatory system through these two sections of the GIT (jejunum and ileum).

### In vivo anti-breast tumor effect of TLNTs

There is growing evidence that the intestinal microbiota plays a critical role in the initiation and development of various diseases [9, 10, 36]. We employed ATBs to investigate whether the intestinal microbiota exerted impacts on the therapeutic outcomes of oral nanomedicines against breast cancer. Mice bearing subcutaneous breast tumors were established and divided into 7 groups, namely the control group, the TLNT (i.v., low)-treated group, the TLNT (i.v., high)-treated group, the TLNT (i.v., high, ATB)-treated group, the TLNT (oral, low)-treated group, the TLNT (oral, high)-treated group and the TLNT (oral, high, ATB)-treated group.

**Fig. 4:**
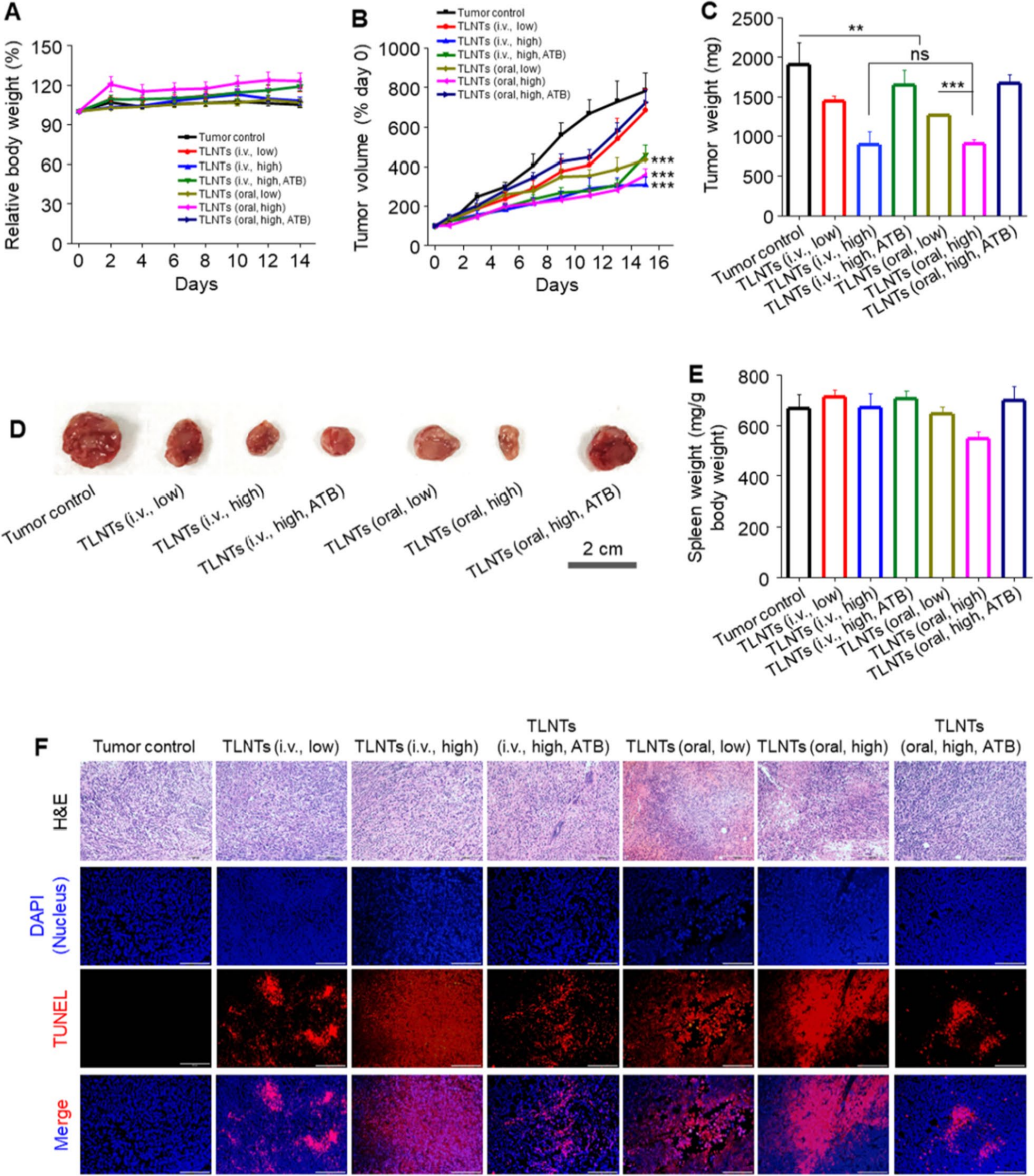
In vivo anti-breast tumor effects of TLNTs. **A** Body weight variations and **B** tumor growth curves of different mouse groups with various treatments during the whole experimental period. **C** Tumor weights, **D** representative tumor images and **E** spleen weights of different mouse groups with various treatments at the end of the experiment. Each point represents the mean ± S.E.M. (n = 5; *p < 0.05, **p < 0.01, ***p < 0.001 and *ns* no significance). **F** H&E- and TUNEL-stained tumor sections showing pathological changes and apoptosis profiles (scale bar: 100 μm)

Body weights and tumor volumes of various mouse groups were recorded during the treatment with TLNTs. Figure 4A showed that there was no apparent difference detected in body weights among the control group and the treatment groups, and all the mice did not exhibit any abnormalities during the entire treatment period. Compared with the control group, the mean tumor volumes were 1.1- and 2.6-fold smaller for the TLNT (i.v.)-treated group and 1.8- and 2.2-fold smaller for the TLNT (oral)-treated group, at a 1.5 and 3 mg/kg protein dose, respectively, on day 15 (Fig. 4B), which are in line with morphological changes in tumor weights and sizes (Fig. 4C, D). Meanwhile, we found that the anti-breast tumor effect of TLNTs (i.v. or oral) greatly decreased after the treatment of broad-spectrum ATBs. It was worth noting that the control group showed negligible variations in spleen weights compared with the TLNT-treated groups, except the TLNT (oral, high)-treated group (Fig. 4E). The spleen weight variation is a result of systemic inflammatory responses [37], and the inflammation is associated with the development of tumorgenesis [38]. Therefore, the strong anti-tumor outcomes of the TLNT (oral, high)-treated group might contribute to the capacity of TLNTs to alleviate the inflammatory responses. These results collectively demonstrate that the TLNT (oral, high)-treated group exhibits comparable anti-tumor effects as the TLNT (i.v., high)-treated group.

Next, we performed a histological assay to evaluate the proliferation profiles of tumor cells. As shown in Fig. 4F, H&E staining images implied that the TLNT treatment caused obvious decreases in the tumor cell amounts in the tumor tissues sections. Moreover, TUNEL staining results indicated that obvious red fluorescence signals were shown in all groups receiving the treatment of TLNTs, particularly in the TLNT (i.v., high)-treated group and the TLNT (oral, high)-treated group, while there was the other way around in the control group (Additional file 1: Fig. S6). These findings reveal that the treatment of TLNTs with high dosages apparently has a stronger capability to cause the apoptosis and retard the growth of tumor cells regardless of the administration approaches (i.v. injection and oral administration).

**Fig. 5:**
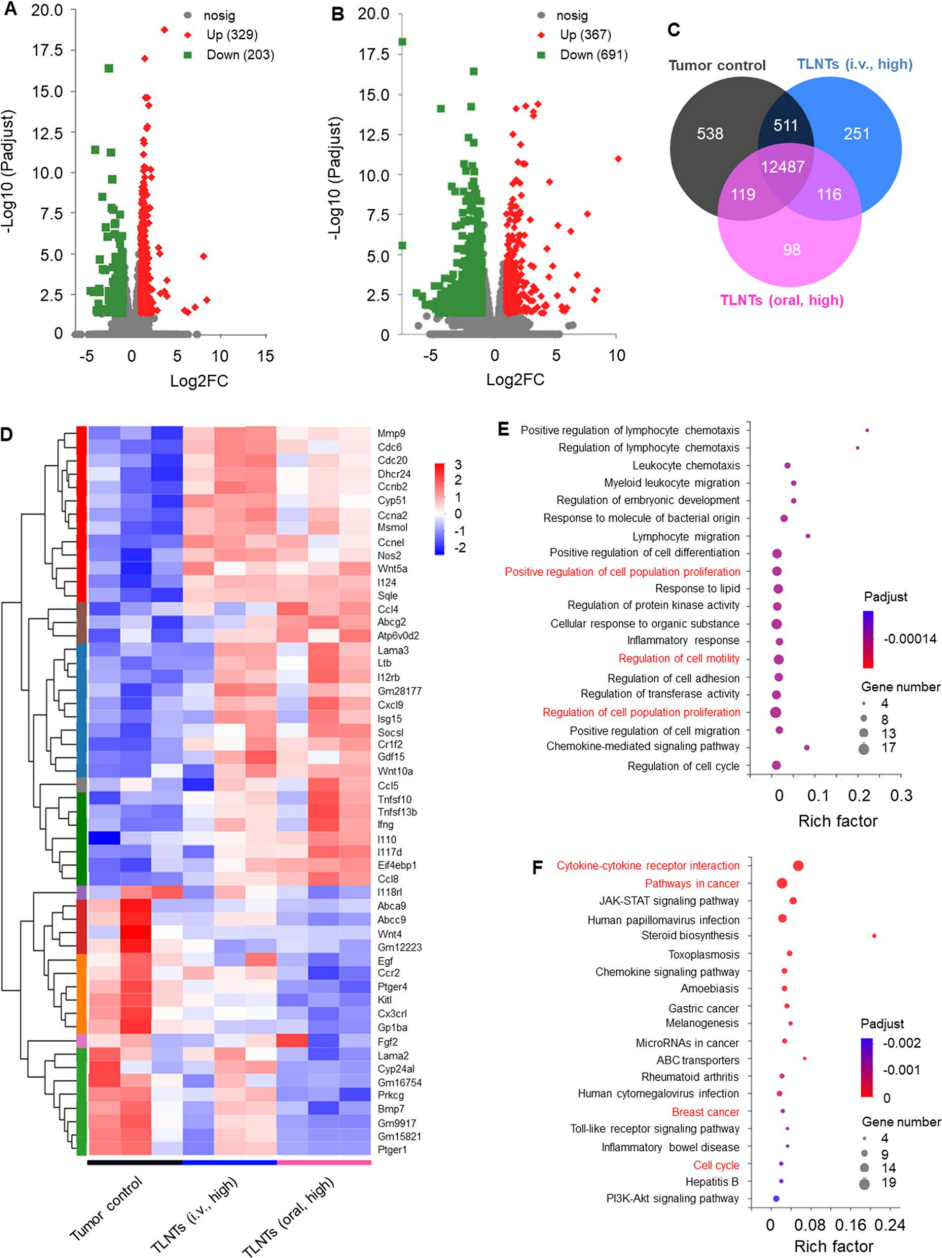
Transcriptome analysis of tumors from various mouse groups. **A** Volcano plot of differentially expressed genes (DEGs) between the control group and the LTNT (i.v., high)-treated group. **B** Volcano plot of DEGs between the control group and the LTNT (oral, high)-treated group. **C** Venn diagram showing overlapped genes among the control group, the LTNT (i.v., high)-treated group and the LTNT (oral, high)-treated group. **D** Heatmap showing significantly up-regulated and down-regulated genes in the tumors from mouse groups receiving various treatments (fold change ≥ 2 and *p* < 0.05). **E** Gene Ontology (GO) enrichment analysis for genes in the brown module. The color represents the adjusted *p*-values, and the sizes of the spots represent the gene numbers. (F) KEGG pathway analysis for cell apoptosis-associated genes (n = 3)

To unravel the tumor inhibition mechanism of TLNTs, the transcriptome analysis of tumor tissues was conducted with assistance from the Majorbio Company. As presented in Fig. 5A, B, in comparison with the control group, 329 up-regulated genes and 203 down-regulated genes were identified in the TLNT (i.v., high)-treated group, and 367 up-regulated genes and 691 down-regulated genes were identified (fold change ≥ 2 and *p* < 0.05) in the TLNT (oral, high)-treated group, respectively. The Venn diagram (Fig. 5C) indicated that the control group shared the similar expression of 12,998 genes with the TLNT (i.v., high)-treated group and 12,606 genes with the TLNT (oral, high)-treated group. We also found that 538, 251 and 98 genes were exclusively expressed in the control group, the TLNT (i.v., high)-treated group and the TLNT (oral, high)-treated group, respectively. Interestingly, principal component analysis (PCA) revealed that genes were differentially expressed in the control group, the TLNT (i.v., high)-treated group and the TLNT (oral, high)-treated group, respectively (Additional file 1: Fig. S7). Our results also revealed that tumoricidal action genes related to anti-tumor immune responses were up-regulated in tumor tissues in the TLNT-treated groups (Fig. 5D). Other mark genes included NOS2, CCL4, CXCL9, and IL-10. The NOS2-encoded inducible nitric oxide synthase (iNOS) that mediated the tumoricidal activity and produced high output nitric oxide were clearly upregulated in the TLNT-treated groups [39]. Likewise, the upregulated CCL4 and CXCL9 genes could suppress tumors by actively recruiting CD8^+^ T cells [40] and regulating immune cell migration, differentiation and activation, correspondingly [41], while IL-10 could exhibit anti-tumor activity by enhancing NK cell activity [42].

To further investigate the biological functions of differentially expressed genes (DEGs), we performed GO analysis by querying each DEG in tumors from different mouse groups against the GO database, leading to the top 20 GO enrichment terms of DEGs. It was found that the responses to regulate cell proliferation occupied the strongest enrichment degree, since it possessed the largest number and was also involved in the regulation of a cell cycle (Fig. 5E), indicating that TLNTs mainly exerted their anti-tumor function through the interferences of cell cycle and cell proliferation. As reported, polyphenols and flavonoids have growth-inhibiting effects on a variety of tumor cells [43], which is consistent with our results (Fig. 4B, C). The Kyoto encyclopedia of genes and genomes (KEGG) pathway analysis reflected that TLNTs exerted anti-proliferation and pro-apoptosis against breast tumor cells mainly through the cellular signal pathways related to the cytokine-cytokine receptor interaction, JAK-STAT and cell cycle (Fig. 5F).

**Fig. 6:**
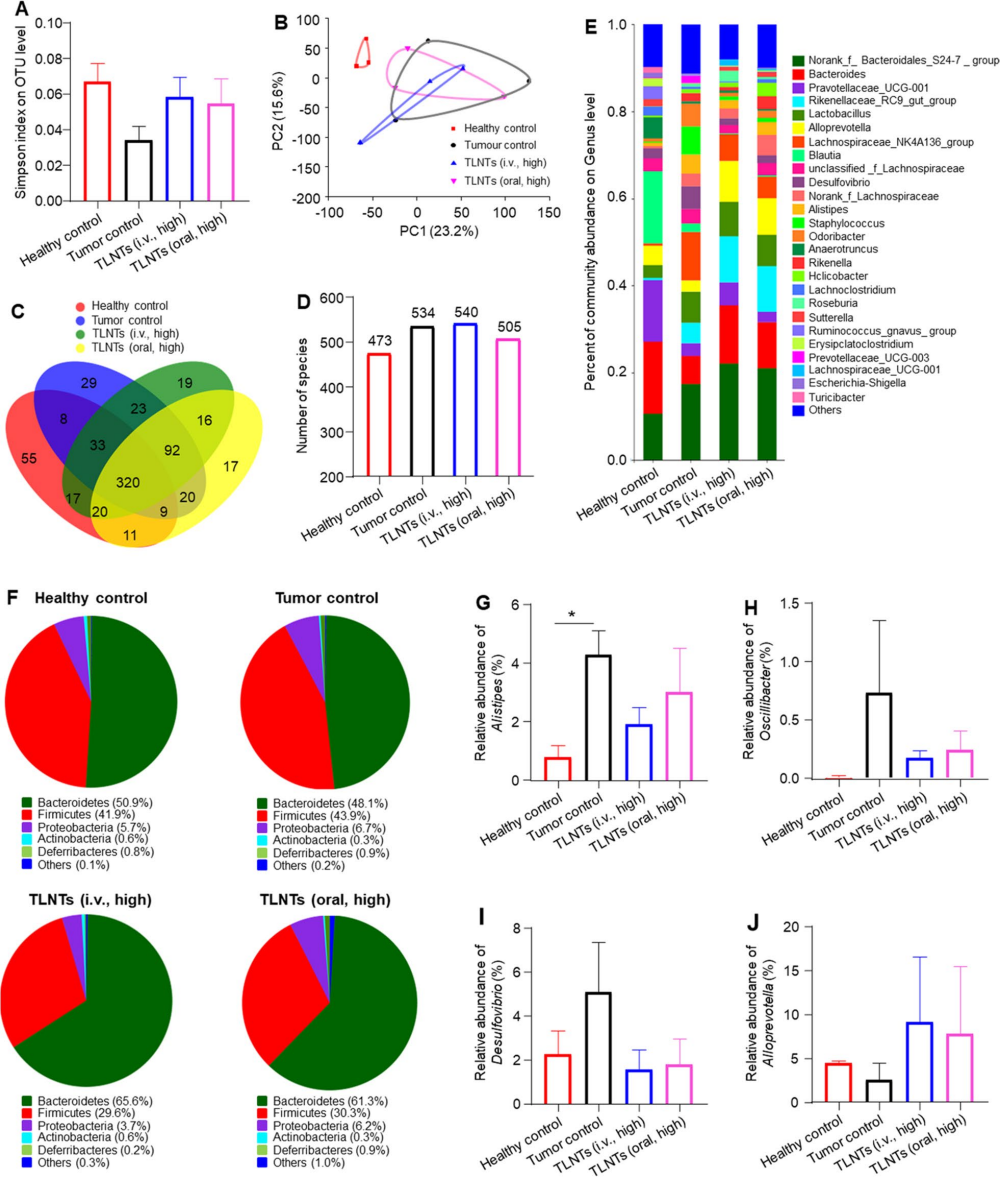
Evaluation of remodeling effects of TLNTs on the intestinal microbiota. **A** *α*-Diversities were presented by box plots of the Simpson indexes. **B** Principal coordinates analysis (PCoA) of the intestinal microbiota. **C** Venn diagram of common and unique bacterial species of mice in each group. (D) Total numbers of microbial species in each group at the OUT level. **E** Relative abundance of intestinal microbiome. Genus-level taxonomy is presented as the percentage of total sequences. **F** Microbial compositions of various mouse groups at the phylum level. **G**–**J** Relative abundance of beneficial bacteria and harmful bacteria in each group. Each point represents the mean ± S.E.M. (n = 3; *p < 0.05)

Accumulating evidence demonstrates that the intestinal microbiota plays a critical role in the development, metastasis and treatment responses of various tumors [44]. Thus, we investigated whether TLNTs could affect the homeostasis of the intestinal microbiota. As shown in Fig. 6A, the *α*-diversity Simpson index reflected the improved diversity of the intestinal microbiota with the treatments of TLNTs (i.v., high and oral, high). Furthermore, principal coordinates analysis (PCoA) suggested that there were dramatic alterations in the microbiome of the tumor control group, in comparison with that of the healthy control group (Fig. 6B). Moreover, Venn diagrams showed that 55, 29, 19 and 17 unique operational taxonomic units (OTUs) were found in the healthy control group, the tumor control group, the TLNT (i.v., high)-treated group and the TLNT (oral, high)-treated group, respectively (Fig. 6C). It was also found that mice bearing breast tumors had increased OUT numbers in their intestinal microbiota compared with the healthy control group (Fig. 6D). To further determine how the TLNT treatments to influence gut microbiota, we analyzed their compositions at the genus level. Figure 6E showed increases in the abundance of the total fecal bacteria for all the TLNT-treated mice. At the phylum level, the statistically significant higher Bacteroidetes/Firmicutes ratios were detected in the TLNT-treated group, compared with the tumor control group (Fig. 6F).

Meanwhile, we found that the abundance of the typical beneficial bacteria and harmful bacteria varied. In particular, *Alistipes*, a potential pathogen, contributed to tumor pathogenesis [45], and its relative abundance greatly decreased in the TLNT (i.v., high)-treated group and the TLNT (oral, high)-treated group, relative to the tumor control group (Fig. 6G). It is documented that *Oscillibacter* has the capacity to elevate the levels of pro-inflammatory cytokines (*e.g.*, IL-1*β* and IL-6), which contribute to the progression of breast tumors [46]. We found that *Oscillibacter* was enriched in the tumor control group, whereas their abundance was significantly decreased in the groups receiving the treatment of TLNTs (i.v., high) and TLNTs (oral, high) (Fig. 6H). Next, compared with the healthy control group, the tumor control group had the increased abundance of *Desulfovibrio*, which was reported to be a cancer-risk genus and could magnify the inflammation and cardiometabolic risks for patients with breast cancer [47] (Fig. 6I). Additionally, it was discovered that the treatments of TLNTs (i.v., high and oral, high) obviously increased the abundance of the beneficial bacteria, *Alloprevotella* (Fig. 6J) [48]. The above findings clearly imply that TLNTs are liable to modulate the intestinal microbiota by increasing the abundance of beneficial bacteria and decreasing the abundance of harmful bacteria, which are consistent with our previous study about the application of tea flower-derived NTs in the treatment of breast cancer via oral route [9]. Interestingly, the treatment of ATBs was found to significantly attenuate the tumor retardation effects of orally administered TLNTs (Fig. 4). It was reported that intestinal microbiome was essential for the activation of anti-tumor immune responses [49]. The weak anti-tumor activity of TLNTs (oral, high, ATB) implies that the abundance and diversity of intestinal microbiota are crucial for potentiating the anti-tumor immunity and further facilitate TLNTs to exert their anti-tumor activity after oral administration.

### In vivo biosafety evaluation of TLTNs

**Fig. 7:**
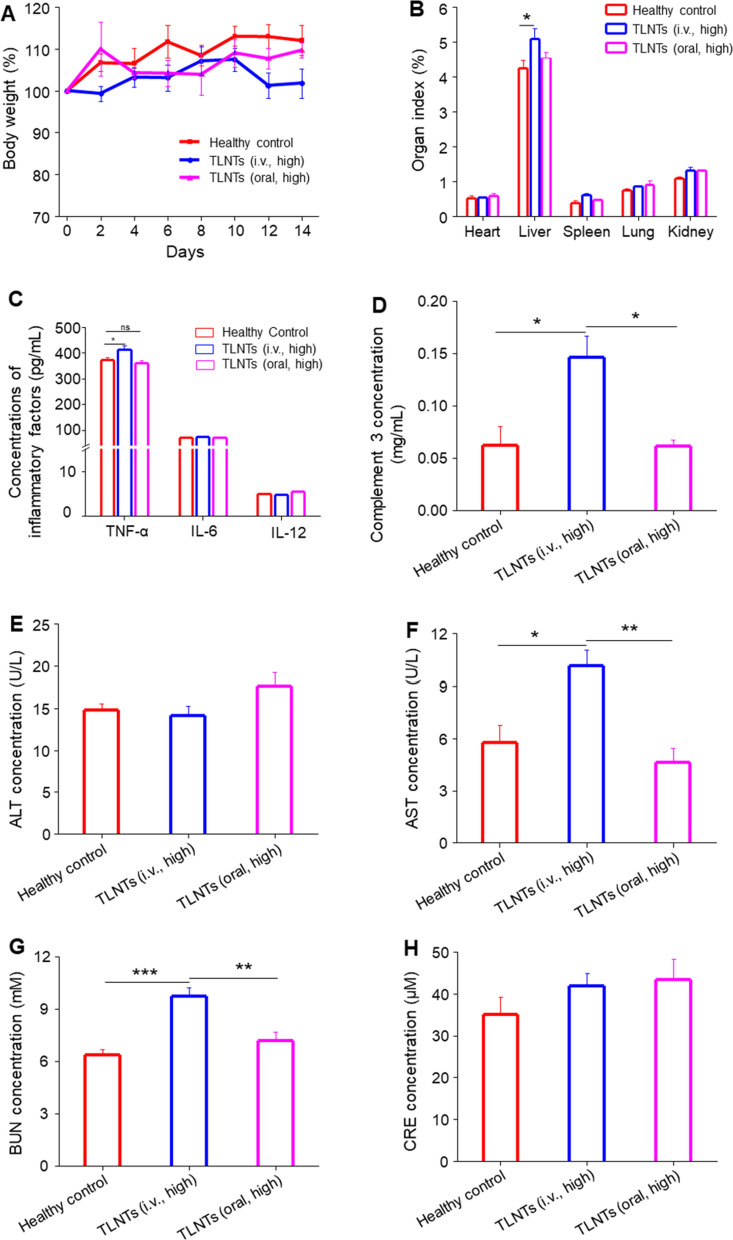
In vivo biosafety evaluation of TLNTs after i.v. injection and oral administration. **A** Body weight variations, **B** organ indices, **C** pro-inflammatory cytokine levels of various mouse groups. The concentrations of **D** Complement C3, **E** ALT, **F** AST, **G** BUN and **H** CRE in plasma from mice receiving the treatment of TLNTs via i.v. and oral routes. Each point represents the mean ± S.E.M. (n = 3; *p < 0.05, **p < 0.01 and ***p < 0.001)

In vivo biosafety of nanomedicines is an essential prerequisite for their medical translation [50] and thus was evaluated. After 4 doses of TLNTs, mouse body weights and organ indexes were recorded. The results indicated that no significant difference was found between the healthy control group and the TLNT (oral, high)-treated groups during the entire experimental period. Strikingly, we visualized that the TLNT (i.v., high)-treated group showed a decrease in body weights (Fig. 7A) and increases in liver indexes and spleen indexes (Fig. 7B), compared with the healthy control group. Although the treatment of TLNTs (i.v., high) did not influence the secreted amounts of pro-inflammatory cytokines (IL-6 and IL-12), this treatment approach resulted in the increased concentrations of the typical inflammatory cytokines (TNF-α) and complement 3, which were obviously higher than those of the healthy control group (Fig. 7C, D).

Nevertheless, we investigated the blood compatibility of TLNTs by a hemolysis assay. It was observed that these TLNTs could not result in hemolysis (Additional file 1: Fig. S8). Moreover, we evaluated the potential toxicity of TLNTs against the liver and spleen by several key indicators, alanine aminotransferase (ALT) and aspartate aminotransferase (AST), two primary indicators of the liver function, as well as urea nitrogen (BUN) and creatinine (CRE), serological indices of the kidney function. We found that the liver and the kidney functioned normally after oral administration of TLNTs, while in the TLNT (i.v., high)-treated group, their hepatorenal function were seen abnormal (Fig. 7E–H). These results demonstrate that TLNTs are, although weak at inducing hemolysis, capable of stimulating the immune system, producing hepatorenal toxicity and altering the hemogram after i.v. injection, suggesting that i.v. route might not be an appropriate approach for administrating plant-derived NTs. However, the systemic cytotoxicities of intravenously injected plant-derived NTs have not been fully elucidated in previous reports [51–54]. In the context of the TLNT (oral, high)-treated group, they showed no obvious variations in terms of body weights, organ indexes, hepatorenal toxicity and complement system activation compared with the healthy control group. These results demonstrate that the plant-derived NTs can be developed as a safe nanoplatform for the treatment of breast cacer via oral administration, rather than i.v. injection.

## Conclusion

Exosome-like nanotherapeutics (NTs) were extracted and purified from fresh tea leaves for breast cancer treatment. These natural NTs were enriched for bio-functional components such as glycolipids, proteins and bioactive small molecules. Cell experiments revealed that over 80% of breast tumor cells could take up TLNTs after co-incubation for 5 h, and the internalized NTs induced the increased amounts of reactive oxygen species, resulting in the mitochondrial damages, the cell cycle arrest and the apoptosis of tumor cells. The subsequent in vivo investigations demonstrated that TLNTs could be mainly absorbed through the small intestine after oral administration to regulate the gene expression profiles in the tumors, modulate the intestinal microbiota and achieve desirable therapeutic outcomes against breast cancer. Notably, oral administration of TLNTs caused no detectable toxicity to the healthy organs, and this therapeutic modality did not induce the activation of the immune system. Overall, the present study exploits a natural ‘green’ nanomedicine for patient-friendly treatment of breast cancer via the oral route.

## Supplementary Information

**Additional file 1. **Experimental details. **Figure S1.** Fluorescence imaging of cellular uptake profiles of DiO-TLNTs by 4T1 cells after co-incubation for 5 h. The scale bar represents 50 μm. **Figure S2.** Quantification of cellular uptake profiles of DiO-TLNTs by 4T1 cells. **A** Flow cytometric histograms, **B** cellular uptake percentages and the corresponding MFI values of 4T1 cells after co-incubation with DiO-TLNTs for 1, 3 and 5 h, respectively. **Figure S3.** Anti-migration capacities of TLNTs. **A** Migration profiles of 4T1 cells after co-incubation with TLNTs for 24 h. **B** Statistical analysis of migration behaviors of 4T1 cells receiving the treatment of TLNTs for 24 h. Each point represents the mean ± S.E.M. (n = 3; **p < 0.01). **Figure S4.** In vitro release profiles of fluorescence dye (DiO) from TLNTs in the stomach stimulating solution (pH 2.5) at 37 °C. Figure S5. Quantification of fluorescence intensities of tumor tissues and major organs at different time points after **A** i.v. injection and **B** oral administration. Data are expressed as mean ± S.E.M. (n = 3). **Figure S6.** Semiquantitative analysis of TUNEL signals of tumor tissues from various treatment groups. Data are expressed as mean ± S.E.M. (n = 3). **Figure S7.** Principle component analysis (PCA) of tumor genes from various mouse groups. **Figure S8.** Blood compatibility evaluation of TLNTs at various protein concentrations. **A** Digital photos and **B** hemolysis rates of erythrocytes receiving the treatment of TLNTs. Each point represents the mean ± S.E.M. (n = 3). **Table S1.** IC_50_ (μg/mL) of TLNTs against various tumor cell lines.

## Data Availability

The datasets generated during and/or analyzed during the current study are available from the corresponding author on reasonable request.

## Abbreviations

AFM: Atomic force microscopy
ALT: Alanine aminotransferase
AST: Aspartate aminotransferase
ATB: Antibiotic
BUN: Urea nitrogen
CRE: Creatinine
CT-26: Mouse colon carcinoma cell line
DAPI: 2-(4-Amidinophenyl)-6-indolecarbamidine dihydrochloride
DCFH-DA: Oxidation-sensitive fluorescent probe
DEGs: Differentially expressed genes
DG: Diacylglycerol
DiO: 3,3ʹ-Dioctadecyloxacarbocyanine perchlorate
DiR: 1,1ʹ-Dioctadecyl-3,3,3ʹ,3ʹ-tetramethylindotricarbocyanine iodide
DLS: Dynamic light scattering
DMEM: Dulbecco’s modified eagle medium
DMSO: Dimethyl sulfoxide
EGCG: Epigallocatechin gallate
GIT: Gastrointestinal tract
GO: Gene ontology
H&E: Hematoxylin and eosin
HPLC-MS/MS: High performance liquid chromatography-tandem mass spectrometry
IC_50_: Half maximal inhibitory concentrations
i.v.: Intravenous
iNOS: Inducible nitric oxide synthase
KEGG: Kyoto encyclopedia of genes and genomes
MCF-7 cell: Human breast cancer cell line
MFI: Mean fluorescence intensity
MGMG: Monogalactosyldiacyglycerol
MTT: Methylthiazolyldiphenyl-tetrazolium bromide
NT: Nanotherapeutic
OTU: Operational taxonomic unit
PA: Phosphatidic acid
PBS: Phosphate buffered saline
PC: Phosphatidylcholine
PCA: Principal component analysis
Pet: Phosphatidylethanol
PI: Propidium iodide
Pme: Phosphatidylmethanol
ROS: Reactive oxygen species
TEM: Transmission electron microscopy
TG: Triglyceride
TLNT: Tea leaf-derived NT
TUNEL: Terminal deoxynucleotidyl transferase-mediated dUTP-biotin nick end labeling
4T1: Mouse breast cancer cell line
