# Supplementary material for: Tea leaf-derived exosome-like nanotherapeutics retard breast tumor growth by pro-apoptosis and microbiota modulation
Source: J Nanobiotechnology. 2023 Jan 4;21:6. doi: 10.1186/s12951-022-01755-5 (PMC9811040; doi:10.1186/s12951-022-01755-5)
Supplement: Supplementary file 1 — Additional file 1. Experimental details. Figure S1. Fluorescence imaging of cellular uptake profiles of DiO-TLNTs by 4T1 cells after co-incubation for 5 h. The scale bar represents 50 μm. Figure S2. Quantification of cellular uptake profiles of DiO-TLNTs by 4T1 cells. A Flow cytometric histograms, B cellular uptake percentages and the corresponding MFI values of 4T1 cells after co-incubation with DiO-TLNTs for 1, 3 and 5 h, respectively. Figure S3. Anti-migration capacities of TLNTs. A Migration profiles of 4T1 cells after co-incubation with TLNTs for 24 h. B Statistical analysis of migration behaviors of 4T1 cells receiving the treatment of TLNTs for 24 h. Each point represents the mean ± S.E.M. (n = 3; **p < 0.01). Figure S4. In vitro release profiles of fluorescence dye (DiO) from TLNTs in the stomach stimulating solution (pH 2.5) at 37 °C. Figure S5. Quantification of fluorescence intensities of tumor tissues and major organs at different time points after A i.v. injection and B oral administration. Data are expressed as mean ± S.E.M. (n = 3). Figure S6. Semiquantitative analysis of TUNEL signals of tumor tissues from various treatment groups. Data are expressed as mean ± S.E.M. (n = 3). Figure S7. Principle component analysis (PCA) of tumor genes from various mouse groups. Figure S8. Blood compatibility evaluation of TLNTs at various protein concentrations. A Digital photos and B hemolysis rates of erythrocytes receiving the treatment of TLNTs. Each point represents the mean ± S.E.M. (n = 3). Table S1. IC50 (μg/mL) of TLNTs against various tumor cell lines. [file 12951_2022_1755_MOESM1_ESM.docx]

**Additional file 1**

**Tea leaf-derived exosome-like nanotherapeutics retard breast tumor growth by pro-apoptosis and microbiota modulation**

Qiubing Chen^a,b,†^, Menghang Zu^a,†^, Hanlin Gong^c,†^, Ya Ma^a^, Jianfeng Sun^d^, Susan Ran^e^, Xiaoxiao Shi^a^, Jinming Zhang^f,*^, Bo Xiao^a,*^

^a^State Key Laboratory of Silkworm Genome Biology, College of Sericulture, Textile and Biomass Sciences, Southwest University, Beibei, Chongqing 400715, China

^b^Chongqing Key Laboratory of Soft-Matter Material Chemistry and Function Manufacturing, School of Materials and Energy, Southwest University, Beibei, Chongqing 400715, China

^c^Department of Integrated Traditional Chinese and Western Medicine, West China Hospital, Sichuan University, Chengdu, Sichuan 610041, China

^d^Botnar Research Centre, Nuffield Department of Orthopedics, Rheumatology and Musculoskeletal Sciences, University of Oxford, Headington, Oxford OX3 7LD, UK

^e^Loomis Chaffee School, Windsor, Connecticut 06095, USA

^f^State Key Laboratory of Southwestern Chinese Medicine Resources, Pharmacy School, Chengdu University of Traditional Chinese Medicine, Chengdu, Sichuan 611137, China

∗ Corresponding author

E-mail addresses: cdutcmzjm@126.com (J. Zhang); bxiao@swu.edu.cn (B. Xiao)

† These authors contributed equally to this work.

**Supplementary experimental details**

**Physicochemical property of** **TLNTs**

Particle size and zeta potential of TLNTs were characterized by DLS (Zeta Sizer Nano-S90, Malvern, UK). The morphologies of TLNTs were imaged by AFM (Seiko Instruments Inc., Chiba, Japan).

Lipidomic analysis of total lipids from TLNTs was performed by Shanghai Cluster Biotechnology Institute (Shanghai, China). In brief, the lipid composition of TLNTs was determined by using a triple quadrupole mass spectrometer (an Applied Biosystems Q-TRAP, Applied Biosystems, Foster City, CA). The data are reported as the percentage of the total signals for the molecular species determined after normalization of the signals to internal standards of the same lipid classes.

Proteins from TLNTs were analyzed by Majorbio BioPharm Technology Co, Ltd (Shanghai, China). Samples proteins were identified and quantified by LC-MS/MS (liquid chromatography coupled with tandem mass spectrometry) using Orbitrap mass spectrometry (Thermo Fisher Scientific, Bremen, Germany). Finally, the data were analyzed based on the National Center for Biotechnology Information (NCBI) database.

To analyze the contents of polyphenol and flavonoid glycosides, TLNTs were dissolved in methanol and centrifuged at 12,000 × g for 10 min, and supernatants were collected. The obtained supernatants were analyzed by high-performance liquid chromatography (HPLC) (Shimadzu Corporation, Kyoto, Japan).

To test in vitro stability of DiO-labeled TLNTs, 20 μM of DiO was incubated with 10 mg of TLNTs at 37 ^o^C for 0.5 h. The labeled TLNTs were passed through ultracentrifuged at 120,000g for 2 h to remove the free dye. DiO-labeled TLNTs and free DiO were suspended in the stomach stimulating solution and added into the dialysis bags (12,000 MWCO, 2 mL). Then dialysis bags were placed at 37 ºC with shaking at 120 rpm. At the pre-determined time intervals, the absorbance of suspensions was measured at 490 nm excitation wavelength and 520 nm emission wavelength using a fluorescence spectrophotometer.

**In vitro cellular uptake profiles of** **TLNTs**

To tract the distribution of TLNTs, they were labeled with a fluorescent lipophilic dye (DiO). In brief, the DiO solution (10 mM) was added to the TLNT suspensions (1 mg protein per 1 mL PBS), and the mixture was incubated for 30 min at 37 ^o^C. The labeled TLNTs were washed 3 times with water to remove the free dye.

4T1 cells were seeded in 12-well plates at a density of 1 × 10^4^ cells/well and incubated overnight. Thereafter, DiO-TLNTs (16 μg/mL) were added to the culture media and incubated with cells. After incubation for 1, 3, and 5 h, respectively, cells were washed with PBS for 3 times to remove the excess DiO-TLNTs. Subsequently, cells were gently detached by using a sterile cell scraper and collected by centrifugation at 1000 × g for 5 min. Finally, cells were re-suspended in PBS and analyzed by flow cytometry (FCM, Beckman Coulter Inc, USA).

4T1 cells were seeded in 12-well plates at a density of 5 × 10^4^ cells/well and incubated overnight. Thereafter, DiO-TLNTs (16 μg/mL) were added to the culture media and incubated with cells for 5 h. Cells were washed with cold PBS for 3 times, and fixed with 4% the paraformaldehyde solution (4%, v/v) for 20 min. Rhodamine B-labeled phalloidin (1:1000 dilution, 500 μL) was added to each well, and cells were incubated for an additional 1 h. Finally, cells were incubated with DAPI for 5 min and imaged using a CLSM (Olympus, FV-3000, Japan).

**TLNTs** **absorption site after oral administration**

DiO-labeled TLNTs were orally administered to BALB/c mice to determine where TFNTs were absorbed in the GIT. Six hours after oral administration, mice were sacrificed, and the entire GIT (stomach, duodenum, jejunum, ileum, and colon) was collected. The different sections of the GIT were embedded into Optimal Cutting Temperature compound, sectioned and image-processed using a CLSM (Olympus, FV-3000, Japan).

**In vitro hemolysis assay of TLNTs**

Briefly, fresh blood was collected from the eyelid posterior sinus vein of mice and centrifuged at 1000 × g for 20 min, washed 3 times and suspended in PBS (2%, v/v). Meanwhile, TLNTs at different concentrations (0.5 to 50 μg/mL protein) were co-cultured with erythrocyte solutions for 1 hour at 37 ^o^C before centrifugation (15 min at 3000 g). Erythrocyte suspensions without treatment were treated as a negative control, and erythrocyte suspensions with the treatment of triton X-100 (1%, w/v) were treated as a positive control. Finally, spectrophotometric tests at 570 nm were used to analyze hemoglobin levels in the supernatants.

**In vivo biosafety of TLNTs**

TLNTs were orally or intravenously administrated to BALB/c mice (6 weeks) at a dose of 3 mg/kg every other day. After 7 doses of TLNTs, mice were sacrificed. The main organs (heart, liver, spleen, lungs and kidneys) and blood were collected for determination of the organ indexes. In the context of plasma, pro-inflammatory factors (TNF-α, IL-6 and IL-12), AST, BUN, CRE and complement C3 were measured using their corresponding kits.


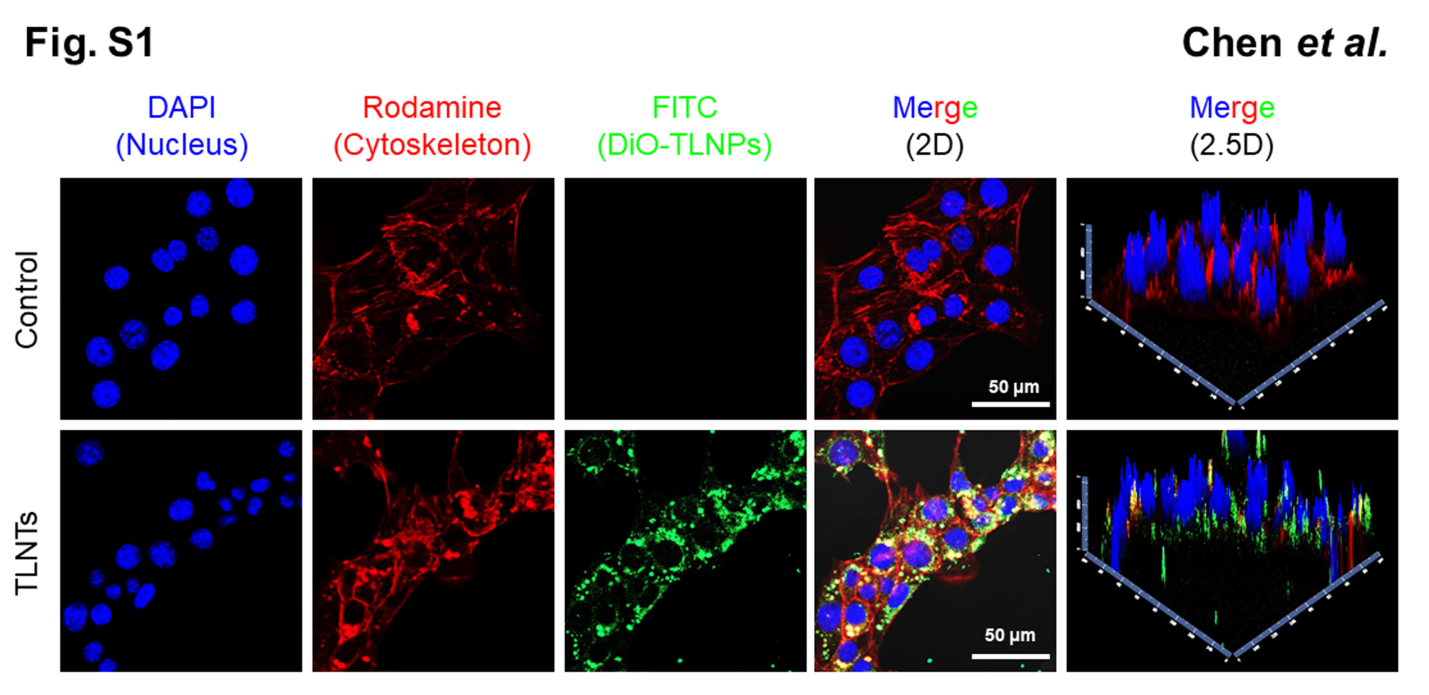


**Figure S1.** Fluorescence imaging of cellular uptake profiles of DiO-TLNTs by 4T1 cells after co-incubation for 5 h. The scale bar represents 50 μm.


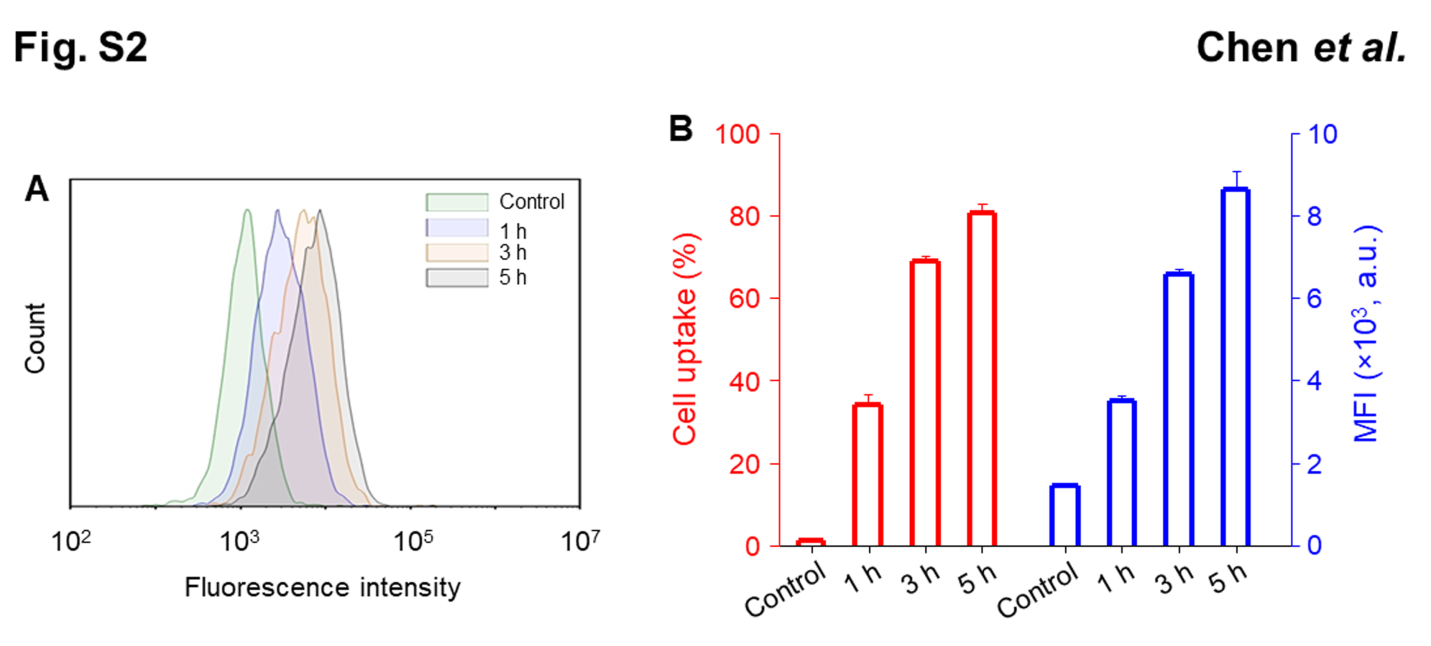


**Figure S2.** Quantification of cellular uptake profiles of DiO-TLNTs by 4T1 cells. (A) Flow cytometric histograms, (B) cellular uptake percentages and the corresponding MFI values of 4T1 cells after co-incubation with DiO-TLNTs for 1, 3 and 5 h, respectively.


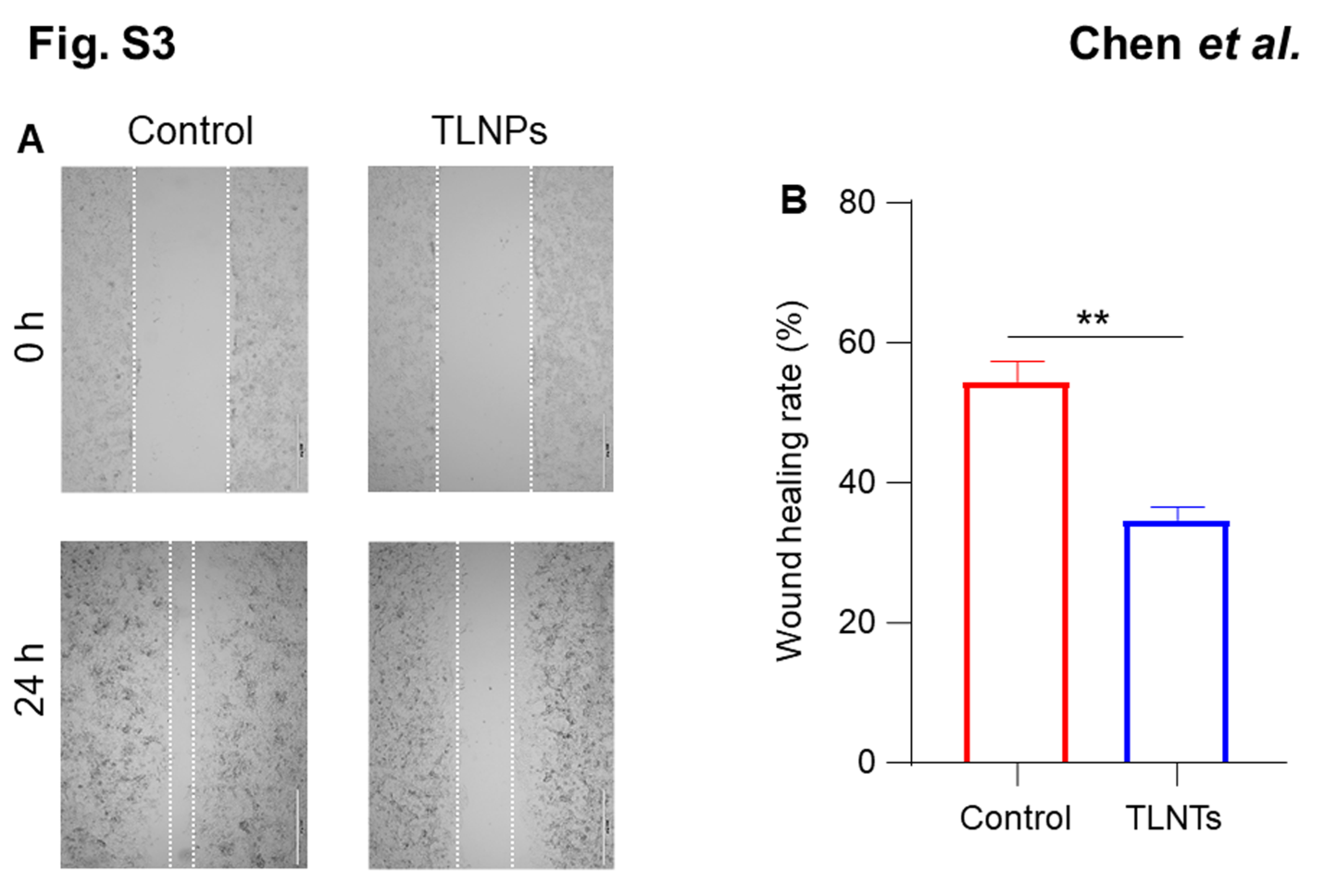


**Figure S3.** Anti-migration capacities of TLNTs. (A) Migration profiles of 4T1 cells after co-incubation with TLNTs for 24 h. (B) Statistical analysis of migration behaviors of 4T1 cells receiving the treatment of TLNTs for 24 h. Each point represents the mean ± S.E.M. (n = 3; ***p* < 0.01).


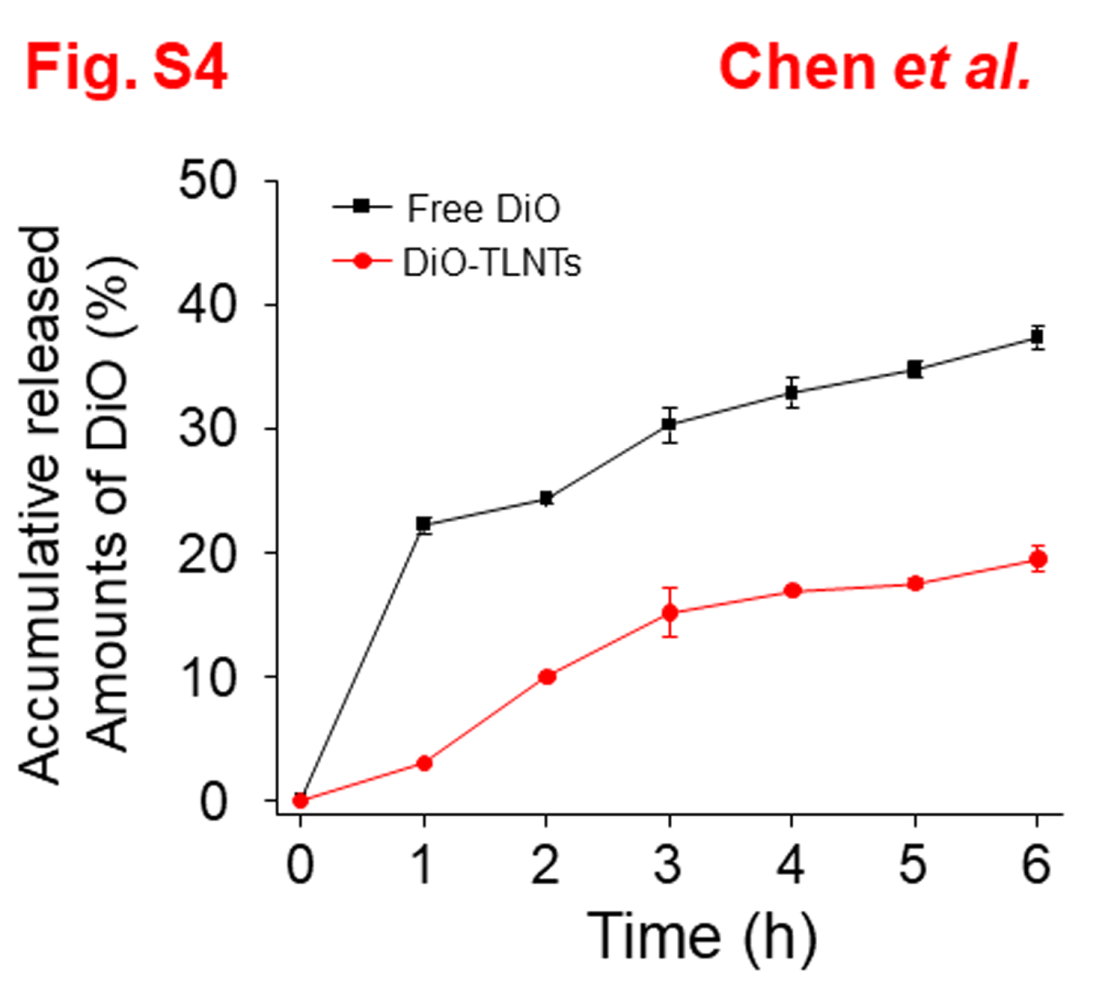


**Figure S4.** In vitro release profiles of fluorescence dye (DiO) from TLNTs in the stomach stimulating solution (pH 2.5) at 37 ^o^C.


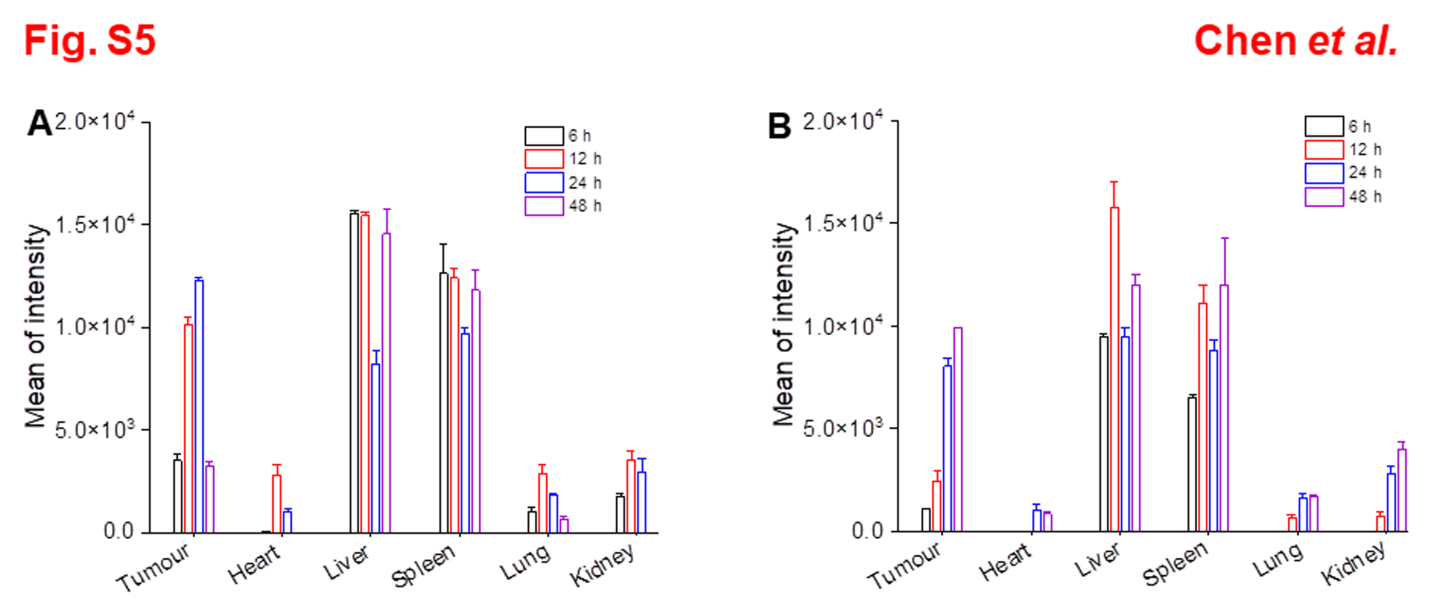


**Figure S5.** Quantification of fluorescence intensities of tumor tissues and major organs at different time points after (A) i.v. injection and (B) oral administration. Data are expressed as mean ± S.E.M. (n = 3).


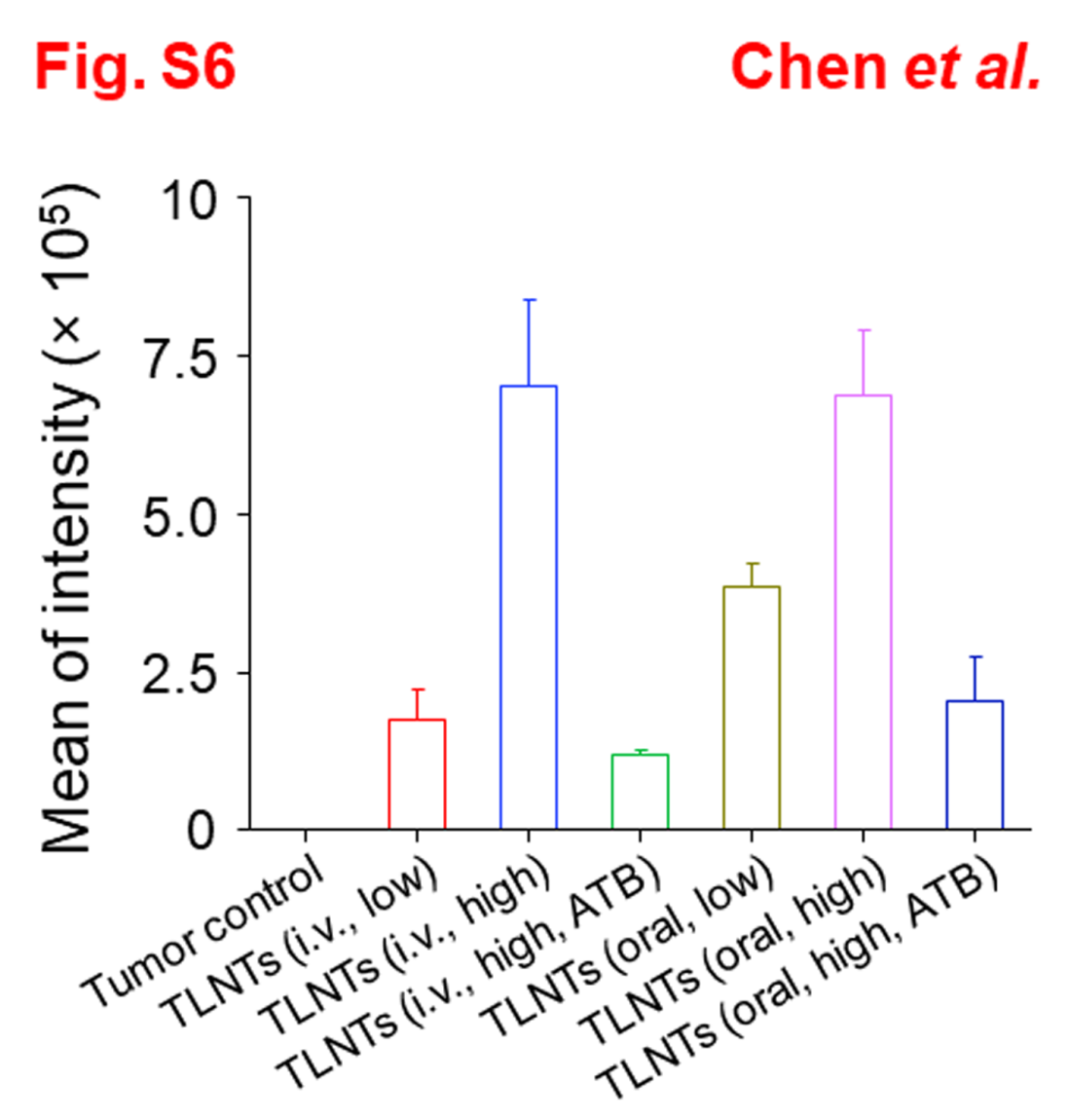


**Figure S6.** Semiquantitative analysis of TUNEL signals of tumor tissues from various treatment groups. Data are expressed as mean ± S.E.M. (n = 3).


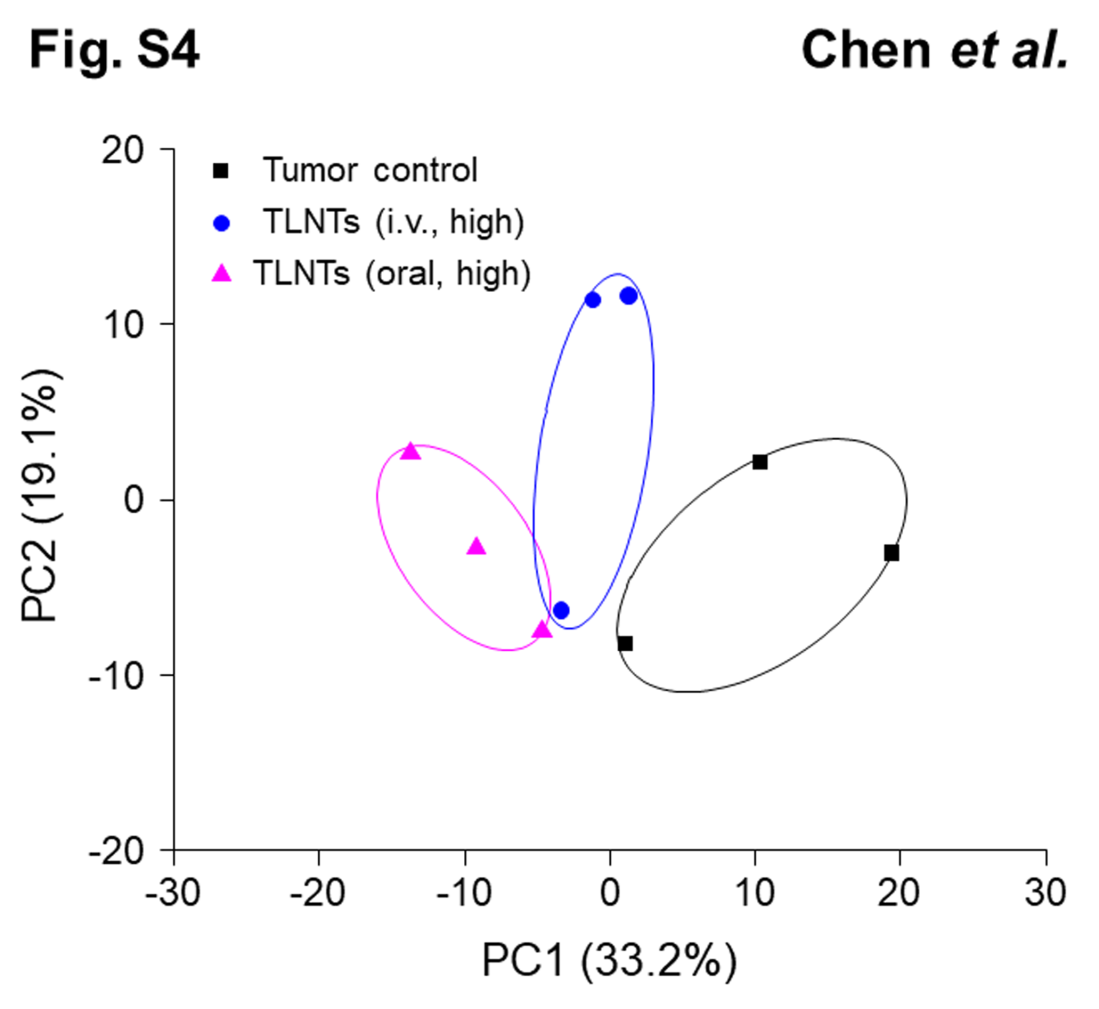


**Figure S7.** Principle component analysis (PCA) of tumor genes from various mouse groups.


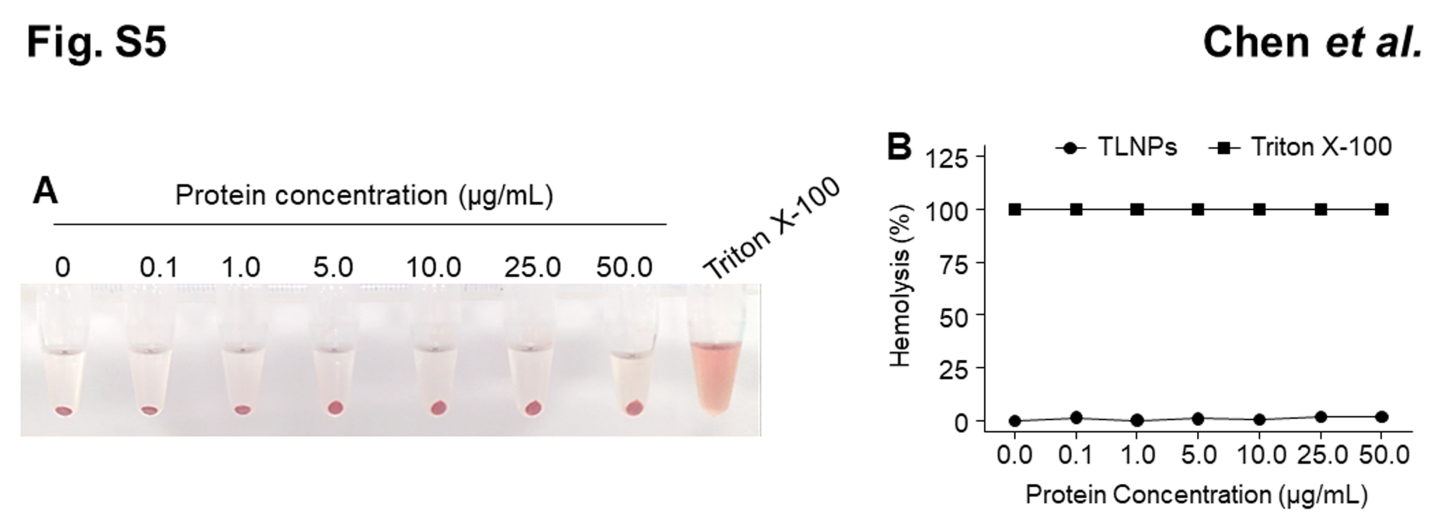


**Figure S8.** Blood compatibility evaluation of TLNTs at various protein concentrations. (A) Digital photos and (B) hemolysis rates of erythrocytes receiving the treatment of TLNTs. Each point represents the mean ± S.E.M. (n = 3).

**Table S1.** IC_50_ (μg/mL) of TLNTs against various tumor cell lines.

| IC_50_ value (μg/mL) | | |
| --- | --- | --- |
|  | 24 h | 48 h |
| CT-26 cells | 43.5 | 5.3 |
| MCF-7 cells | 35.1 | 2.1 |
| 4T1 cells | 23.6 | 0.024 |
